# SARS-CoV-2 evolution influences GBP and IFITM sensitivity

**DOI:** 10.1073/pnas.2212577120

**Published:** 2023-01-24

**Authors:** Dejan Mesner, Ann-Kathrin Reuschl, Matthew V. X. Whelan, Taylor Bronzovich, Tafhima Haider, Lucy G. Thorne, Roberta Ragazzini, Paola Bonfanti, Greg J. Towers, Clare Jolly

**Affiliations:** ^a^Division of Infection and Immunity, University College London, WC1E 6BT London, UK; ^b^Centre for Genomics and Child Health, Blizard Institute, Queen Mary University of London, E1 2AT London, UK; ^c^Epithelial Stem Cell Biology and Regenerative Medicine Laboratory, The Francis Crick Institute, London NW1 1AT, UK

**Keywords:** SARS-CoV-2, GBP, IFITM, restriction, spike

## Abstract

SARS-CoV-2 spike requires proteolytic processing for viral entry. A polybasic furin-cleavage site (FCS) in spike, and evolution toward an optimized FCS by dominant variants of concern (VOCs), are linked to enhanced infectivity and transmission. Here we show interferon-inducible restriction factors Guanylate-binding proteins (GBP) 2 and 5 interfere with furin-mediated spike cleavage and inhibit the infectivity of early-lineage isolates Wuhan-Hu-1 and VIC. By contrast, VOCs Alpha and Delta escape restriction by GBP2/5 that we map to the spike substitution D614G present in these VOCs. Despite inhibition of spike cleavage, these viruses remained sensitive to plasma membrane IFITM1, but not endosomal IFITM2 and 3, consistent with a preference for TMPRSS2-dependent plasma membrane entry. Strikingly, we find that Omicron is unique among VOCs, being sensitive to restriction factors GBP2/5, and also IFITM1, 2, and 3. Using chimeric spike mutants, we map the Omicron phenotype and show that the S1 domain determines Omicron’s sensitivity to GBP2/5, whereas the S2’ domain determines its sensitivity to endosomal IFITM2/3 and preferential use of TMPRSS2-independent entry. We propose that evolution of SARS-CoV-2 for the D614G substitution has allowed for escape from GBP restriction factors, but the selective pressures on Omicron for spike changes that mediate antibody escape, and altered tropism, have come at the expense of increased sensitivity to innate immune restriction factors that target virus entry.

## Significance

GBPs and IFITMs are potent innate immune restriction factors that can inhibit viral infectivity. Specifically, GBPs perturb furin-mediated processing of viral envelope proteins, targeting viruses that rely on proteolytic processing for optimal infectivity. Here, we report that GBP2 and GBP5 inhibit the cleavage of SARS-CoV-2 spike and reduce viral infection. Notably, while the infectivity of early-lineage SARS-CoV-2 isolates is restricted by GBP2/5, VOCs Alpha and Delta have evolved to escape this inhibition. By contrast, Omicron is sensitive to inhibition by GBP2/5, as well as inhibition by endosomal IFITM2 and IFITM3 consistent with Omicron’s use of alternative cell entry pathways. Our data show how VOC evolution under different selective pressures has influenced sensitivity to antiviral restriction factors, and thus, innate immunity.

SARS-CoV-2 infects cells by binding of the viral spike (S) protein to the angiotensin-converting enzyme 2 (ACE2) receptor on host cells (1). For fusion to proceed after ACE2 binding, spike must be cleaved by host cell proteases to become activated and fusion-competent. In this step-wise process, spike is preprocessed by furin-like proteases in virus-producing cells at the S1/S2 junction (2, 3), followed by a second cleavage event at the S2’ site mediated by TMPRSS2 protease at the target cell surface (1), releasing the fusion peptide and allowing for viral fusion at the plasma membrane. Alternatively, TMPRSS2-independent endocytic uptake can occur in some cell types resulting in spike being cleaved and activated for fusion by endosomal cathepsin proteases (2). The polybasic furin cleavage site (FCS) Arg–Arg–Ala–Arg (or RRAR) motif that is targeted by furin is absent in closely related coronaviruses, including the closest relatives of SARS-CoV-2, bat RaTG13 and pangolin CoV (2, 4, 5). This has led to the notion that presence of an FCS in the SARS-CoV-2 ancestor was associated with successful zoonosis and pandemic transmission between humans. In support, the FCS is required for efficient proteolytic cleavage of SARS-CoV-2 spike (2), virus infection of human airway cells (1, 2, 6, 7), cell–cell fusion and syncytia formation (2, 8, 9), and transmission (6, 7).

Following the identification of the first SARS-CoV-2 strain circulating in humans (Wuhan-Hu-1), several variants of concern (VOCs) have emerged, each containing a constellation of mutations and further adaptations to host that have been associated with increased transmission. These major previous and current VOCs are designated Alpha (PANGO lineage B.1.1.7), Beta (B.1.351), Gamma (P1), Delta (B.1.617.2), and latterly Omicron (B.1.1.529). Of these, Alpha, Delta, and Omicron have been the most successful globally, each rapidly replacing the previous dominating VOC over time (Omicron > Delta > Alpha). These VOCs have an increasing number of nonsynonymous mutations in spike, which alter entry efficiency and kinetics (10) and enhance immune escape, including innate immunity (11–13). Much focus has been on spike mutations arising from selective pressure for antibody escape; however, SARS-CoV-2 spike continuously adapts in other ways to the human host. For example, dominant VOCs (Alpha, Delta, and Omicron) harbor mutations near and within the FCS, which enhances spike cleavage, indicative of evolution toward an optimized FCS (14, 15).

Successful viral replication and transmission requires evasion or antagonism of host defensive processes, notably innate immunity, and is particularly important for zoonotic viruses that must adapt quickly or be suitably preadapted to the new host. Innate immune activation up-regulates host cell proteins termed restriction factors that target key steps in viral replication to limit and control infection. Guanylate-binding proteins (GBP) are type-I and type-II interferon-stimulated genes (ISGs) and a subfamily of guanosine triphosphatases (GTPases) that can act as intracellular antiviral restriction factors (16). GBP2 and 5 potently inhibit furin-mediated processing of viral envelope proteins, inhibiting infection of HIV-1, Influenza A Virus, Zika, and measles viruses, all of which require furin cleavage for optimal infectivity (16–19). Notably, GBPs are up-regulated in airway epithelial cells and during SARS-CoV-2 infection (20, 21). Thus, GBPs comprise a key effector of the antiviral innate immune response that can act to limit infectious virus production during replication. Likewise, the interferon-induced transmembrane (IFITM) protein family also acts broadly to block viral entry, inhibiting viral fusion with cellular membranes, including SARS-CoV-2 (7, 12, 21–24).

Here, we investigated the capacity of GBP2 and 5 to inhibit SARS-CoV-2 spike cleavage and virus infectivity, and tested whether evolution of VOCs has led to escape from GBPs. We find differential sensitivity of SARS-CoV-2 spikes to GBP indicative of independent adaptation to host. We also test the consequence of GBP inhibition of furin-cleavage on SARS-CoV-2 sensitivity to another family of entry-targeting restriction factors, IFITMs. Notably, while Alpha and Delta have evolved to escape restriction by GBPs through the D614G substitution in spike, we find that Omicron is uniquely sensitive to inhibition GBP2/5 and also endosomal IFITM2 and 3, consistent with Omicron evolving under different selective pressures, driving increased spike mutations that alter spike activity, cell entry, and tropism.

## Results

### GBP2 and 5 Inhibit Wuhan-Hu-1 and Omicron, but not Alpha and Delta Spike-Mediated Infectivity.

**Fig. 1.**
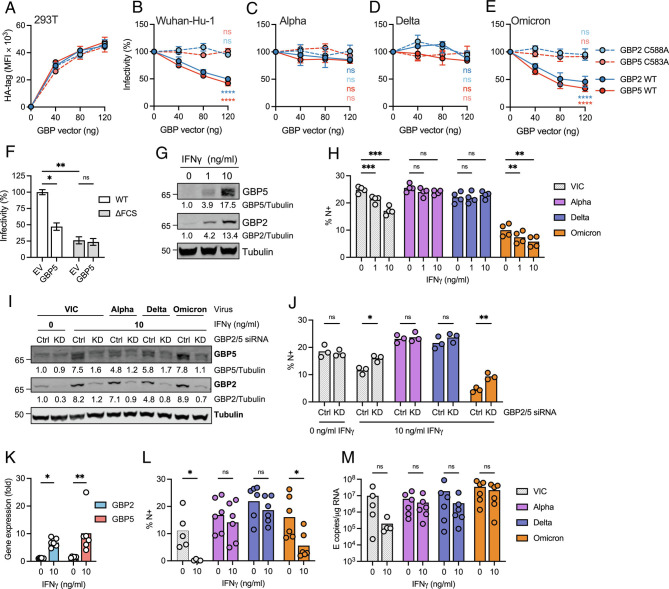
GBP2 and 5 inhibit Wuhan-Hu-1 and Omicron, but not Alpha and Delta spike-mediated infectivity. (*A*) Expression of HA-tagged GBPs in pseudovirus (PV)-producing 293T cells measured by flow cytometry. (*B*–*E*) Infectivity of PVs produced by 293T cells in the presence of increasing amounts of plasmid encoding GBP2, GBP2 C588A, GBP5, or GBP5 C583A measured by luciferase assay (RLU) on Caco2 cells. Shown are percentage infectivity of PV made in the presence of GBPs normalized to empty vector (EV) control (no GBP, set to 100%). Percent infectivity of (*B*) Wuhan-Hu-1, (*C*) Alpha, (*D*) Delta, and (*E*) Omicron spike PV infection is shown. Shown are the mean ± SEM from three independent experiments. (*F*) Infectivity of WT and ΔFCS Wuhan-Hu-1 spike PV made in the presence of 120 ng GBP5 or EV control and titrated on Caco2 cells. Shown is percent infectivity (±SEM from three independent experiments) normalized to WT spike EV control. (*G*, *H*) Calu-3 cells were treated with indicated doses of IFNγ for 8 h prior to infection with indicated SARS-CoV-2 variants for 36 h. (*G*) Cell lysates were immunoblotted for GBP2, GBP5, and tubulin. Quantification shows relative expression of GBP2/5 over tubulin and normalized to untreated control. (*H*) Equal doses (E copies/cells) of SARS-CoV-2 virus produced in cells from (*G*) were used to infect Caco2 cells, and infection levels were determined at 24 hpi by intracellular staining for nucleocapsid (N) protein. Percentage positive cells are shown (% N+ cells). Bars show the mean and individual values from two independent experiments. (*I*, *J*) Calu-3 cells were pretreated with combined GBP2 and GBP5 siRNA (KD) or nontargeting control (Ctrl, 120 and 32 h preinfection) and indicated doses of IFNγ (8 h preinfection) before infection with indicated SARS-CoV-2 variants for 36 h. (*I*) Cell lysate immunoblots for GBP2, GBP5, and tubulin. Quantification shows relative expression of GBP2/5 over tubulin and normalized to Ctrl siRNA IFNγ-untreated control. (*J*) Equal doses (E copies/cells) of SARS-CoV-2 virus produced in cells from (*I*) were used to infect Caco2 cells for 24 h and infection was quantified by N protein staining (% N+ cells). Bars show the mean and replicate values from one experiment. (*K*–*M*) Primary human airway epithelial cells (HAEs) were treated with indicated doses of IFNγ for 12 h prior to infection with equal doses (1,500 E copies/cell) of indicated SARS-CoV-2 variants for 72 h. (*K*) GBP2 and GBP5 gene expression (fold increase over 0ng/mL IFNγ) at 72 hpi. (*L*) Virus release from infected HAE was collected in apical washes at 72 hpi, equal volumes were used to infect Caco2 cells for 24 h, and infection was quantified by N protein staining (% N+ cells). (*M*) Intracellular replication in HAE measured by E copies at 72 hpi. Two-way ANOVA (*B*–*F*, *H*, *J*–*M*) with Dunnett’s posttest was used. (*B*–*E*) Statistical significance for GBPs (120 ng) compared EV control is indicated. FCS, furin cleavage site. ns, not significant; *P < 0.05; **P < 0.01; ***P < 0.001; ****P < 0.0001.

To determine whether the antiviral restriction factors GBP2 and 5 have activity against SARS-CoV-2, a pseudovirus (PV) assay was used in which SARS-CoV-2 spike is incorporated into lentiviral particles (herein termed PV) (*SI Appendix*, Fig. S1*A*). This allows direct comparison of how evolution of amino acid changes in spike alone (*SI Appendix*, Fig. S1*B*) has influenced GBP sensitivity, without confounding contributions of other SARS-CoV-2 variant proteins on infectivity. 293T cells were cotransfected with plasmids encoding SARS-CoV-2 spike, lentiviral genome, and increasing doses of GBP-expressing plasmid. GBP expression was confirmed by flow cytometry staining for the HA-tag and immunoblotting (Fig. 1*A* and *SI Appendix*, Fig S2 *A* and *B*). Fig. 1*B* shows that Wuhan-Hu-1 PV made in the presence of GBP2 or 5 was significantly less infectious (50%) when titrated onto Caco2 target cells, with both GBP2 and 5 inhibiting PV infectivity in a dose-dependent manner (Fig. 1*B* and *SI Appendix*, Fig. S2*C*). We selected naturally permissive intestinal epithelial Caco2 cells as targets for PV infection for their endogenous expression of both ACE2 and TMPRSS2 (25, 26). As expected, the isoprenylation-deficient mutants of GBP2 (GBP2 C588A) and GBP5 (GBP5 C583A), which are mislocalized in the cell and lose antiviral activity against other viruses such as HIV-1 (17, 18) (*SI Appendix*, Fig. S2 *G* and *H*), showed no inhibitory activity against Wuhan-Hu-1 (Fig. 1*B*). Inhibition of Wuhan-Hu-1 PV infectivity was not due to lack of spike expression on 293T cells since expressing GBPs did not alter plasma membrane levels of spike measured by flow cytometry (*SI Appendix*, Fig. S3*B*). Immunofluorescence imaging of spike in 293T cells revealed a similar pattern of diffuse and punctate spike staining in the presence and absence of GBP5, and a small but significant increase in spike colocalization with the ER marker calnexin in the presence of GBP5 (*SI Appendix*, Fig. S3 *C*–*E*). Strikingly, and in contrast to Wuhan-Hu-1, PV particles containing the Alpha and Delta spikes were completely resistant to GBP2 and 5 restriction and showed no loss of PV infectivity (Fig. 1 *C* and *D* and *SI Appendix*, Fig. S2 *D* and *E*). By contrast, PVs containing the Omicron BA.1 (Omicron) spike behaved like Wuhan-Hu-1 and were sensitive to restriction by GBP2 and 5, evidenced by a significant 60% loss of infectivity (Fig. 1*E* and *SI Appendix*, Fig. S2*F*).

To measure the contribution of the SARS-CoV-2 furin-cleavage site to PV infectivity, and thus determine the maximum loss of infectivity that be expected if spike processing is completely prevented (comparing to the effects of GBP2/5), we used a Wuhan-Hu-1 spike in which the FCS has been deleted (ΔFCS) to prevent spike cleavage (7). Deletion of the FCS resulted in a significant 75% reduction in PV infectivity, when compared with wild-type Wuhan-Hu-1 spike (Fig. 1*F*). This was not further reduced by GBP5 expression (Fig. 1*F*). Consistent with the loss of Wuhan-Hu-1 PV infectivity being mediated by GBP2/5 inhibition of furin cleavage (and not off-target effects), no difference in infectivity was seen when PV were titrated onto Vero.E6 cells (*SI Appendix*, Fig. S2*I*) that do not require furin-processing for SARS-CoV-2 infection, as spike is processed by endosomal cathepsins (2, 27). Furthermore, GBP2/5 expression also significantly reduced infectivity mediated by MERS-CoV spike, which contains a furin-cleavage site (28), but not by SARS-CoV-1 spike that lacks a furin-cleavage site (*SI Appendix*, Fig. S2 *J* and *K*).

Next, we tested whether GBP2/5 also inhibited the infectivity of live, replication competent SARS-CoV-2 isolates. To do this, Calu-3 cells were pretreated with IFNγ to up-regulate expression of GBP2/5 (Fig. 1*G*) (16, 18) and then infected with SARS-CoV-2 isolates: VIC (an early-lineage, Wuhan-like isolate) and VOCs Alpha, Delta, and Omicron (BA.1). Virus-containing supernatants were harvested from Calu-3 infections and used to infect Caco2 target cells. Input doses were equalized by viral E gene copies and nucleocapsid-positive (N+) Caco2 cells were quantified by flow cytometry. We observed a significant reduction in the infectivity of VIC and Omicron viruses produced by IFNγ-treated Calu-3 cells (that was dose dependent) when compared with virus produced by Calu-3 cells not treated with IFNγ (Fig. 1*H* and *SI Appendix*, Fig. S4*A*). By contrast, Alpha and Delta viruses showed no loss of infectivity (Fig. 1*H*), a finding consistent with our PV assay data. Importantly, depleting GBP2 and GBP5 with siRNA in IFNγ-treated Calu-3 cells (Fig. 1*I*) completely rescued the infectivity of GBP-sensitive isolates VIC and Omicron (Fig. 1*J* and *SI Appendix*, Fig. S4*B*) confirming that the antiviral effects of IFNγ on reducing virus infectivity in producer cells is mediated by GBP2/5. We note the presence of an additional band detected by the GBP5, but not GBP2 antibody in IFNγ-treated Calu-3 cells, whether this represents a posttranslational modification of GBP5 or a GBP5 splice variant for example remains unclear. Concordant with our Calu-3 results, virus recovered from infections of primary human airway epithelial (HAE) cells, treated with IFNγ to up-regulate GBP2/5, showed a significant loss of infectivity of VIC and Omicron, but not Alpha and Delta viruses, despite all VOCs showing similar infection levels in HAE (Fig. 1 *K*–*M* and *SI Appendix*, Fig. S4*C*). Collectively these data reveal that VOCs Alpha and Delta have evolved spikes that escape restriction factors GBP2 and 5 that inhibit early-lineage SARS-CoV-2 isolates Wuhan-Hu-1 and VIC, whereas Omicron has not.

### GBP2 and 5 Inhibit SARS-CoV-2 Spike Cleavage.

**Fig. 2.**
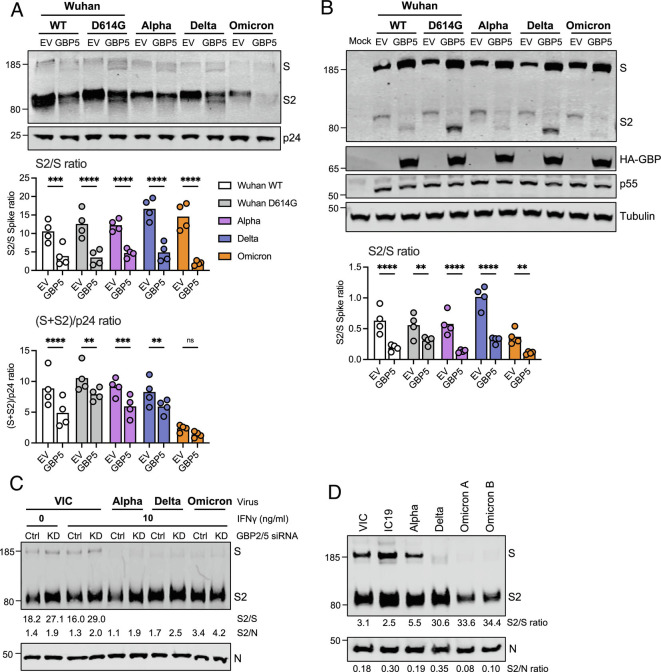
GBP2 and 5 inhibit SARS-CoV-2 spike cleavage. (*A*, *B*) Spike PVs were produced in 293T cells in the presence of 80 ng GBP plasmid or empty vector (EV) control. PVs and producer cell lysates were immunoblotted for spike, lentiviral Gag (p24 and p55), GBP (HA-tag) and tubulin. (*A*) Immunoblot of VOC spike PV produced in the presence of GBP5 or EV. A representative immunoblot is shown. Graphs show quantification pooled from four independent experiments, measuring the proportion of cleaved spike in PV (S2/S) (*Top*) and total spike incorporation (S+S2/p24) (*Bottom*). Mean and individual values are shown. (*B*) Producer 293T cell lysate from (*A*). (*C*) Calu-3 cells were pretreated with GBP2 and GBP5 siRNA (KD) or nontargeting control (Ctrl) and indicated doses of IFNγ before infection with indicated SARS-CoV-2 variants for 36 h as described in Fig. 1. Equal doses (E copies) of clarified viruses were sucrose-purified and immunoblotted for spike (S) and nucleocapsid (N) protein. Quantification shows proportion of spike cleavage (S2/S) and cleaved spike incorporation into virions (S2/N). (*D*) Indicated SARS-CoV-2 variants were produced in Caco2 cells, and equal doses of virus (E copies) were immunoblotted for S and N protein. Quantification shows proportion of cleaved spike (S2/S) and cleaved spike incorporation into virions (S2/N). Two independent Omicron BA.1 isolates (*A* and *B*) are shown. Two-way ANOVA with Dunnett’s posttest (*A*, *B*) was used. ns, not significant; *P < 0.05; **P < 0.01; ***P < 0.001; ****P < 0.0001.

Given spike is cleaved by furin, and that furin is inhibited by GBPs, we next measured the effects of GBP2 and 5 on spike S1/S2 cleavage. Visualizing uncleaved (S) and cleaved spikes (S2) by immunoblotting of purified PV particles (Fig. 2*A* and *SI Appendix*, Fig. S5*A*) and cell lysates (Fig. 2*B* and *SI Appendix*, Fig. S5*B*) revealed clear differences in the processing of Wuhan-Hu-1 spike in the presence of GBP2 and 5, compared with either no GBPs, or the inactive mutants GBP2 C588A and GBP5 C583A (*SI Appendix*, Fig. S5 *A* and *B*). Quantifying this across all spike variants from four independent experiments showed that GBP5 significantly reduced spike cleavage (lower S2/S ratio) and incorporation (S+S2/p24) into Wuhan-Hu-1, Alpha, Delta, and Omicron PV particles (Fig. 2*A*). A significant spike cleavage defect was also apparent when immunoblotting cell lysates (Fig. 2*B*), consistent with GBP perturbation of intracellular spike processing during PV production. Having observed that the S2 spike subunit migrated at a lower molecular weight in the presence of GBP, we sought to test whether this may reflect a change in spike glycosylation. A similar alteration in HIV-1 envelope (Env) glycosylation has been reported when furin-cleavage is inhibited by GBPs, presumably due to differences in the intracellular trafficking of uncleaved versus cleaved Env influencing protein glycosylation (17, 18). *SI Appendix*, Fig. S5*C* shows that PNGase treating 293T cell lysates abolished the spike band shift, such that the S2 subunit migrated at the same molecular weight +/− GBP. Taken together, these data indicate that GBP expression inhibits furin cleavage and also influences spike N-linked glycosylation (29, 30) resulting in reduced particle infectivity.

Quantifying spike cleavage (S2/S) in virus produced by IFNγ-treated Calu-3cells was more challenging due to low levels of uncleaved spike detected in VOC particles (Fig. 2*C*) and here immunoblotting cell lysates did not show an obvious cleavage defect (*SI Appendix*, Fig. S5*D*), unlike PV assays. However, quantifying spike cleavage by immunoblotting purified VIC viral particles (from the experiment shown in Fig. 1 *I* and *J*) revealed that RNAi depletion of GBP2/5 in IFNγ-treated Calu-3 increased the amount of cleaved spike product in virions (S2/S) (Fig. 2*C*), although this effect was not as striking as what was seen in PV assays. The observation that GBP2/5 knockdown also increased VIC spike cleavage in Calu-3 cells that were not treated with IFNγ (Fig. 2*C*, 0ng/mL IFNγ condition) is explained by the presence of basal levels of inhibitory *GBP2/5* expression in these cells (Fig. 1*I* and *SI Appendix*, Fig. S5*D*) that was also depleted using RNAi.

Alpha, Delta, and Omicron spikes contain optimizing mutations in the FCS that have been reported to enhance S1/S2 cleavage (14, 15) (P681H, P681R and P681H/N679K/H655Y respectively). Consistent with this, immunoblotting of purified viral isolates (produced in the absence of GBPs) showed a spike cleavage hierarchy of Omicron > Delta > Alpha > IC19/VIC (Fig. 2*D*); however, Omicron’s optimized FCS does not protect from GBP restriction (Fig. 1*E*). These data, as well as our observation that both Alpha and Delta are resistant to GBP-mediated restriction of viral infectivity despite GBPs inhibiting spike cleavage, suggests that evolution of VOCs Alpha and Delta for escape from GBPs cannot simply be attributed to the presence of optimized FCS. This implicates other spike adaptations, beyond the FCS, in allowing Alpha and Delta to overcome GBP restriction of infectivity.

### D614G Substitution in Alpha and Delta Spikes Confers Resistance to GBP Restriction.

**Fig. 3.**
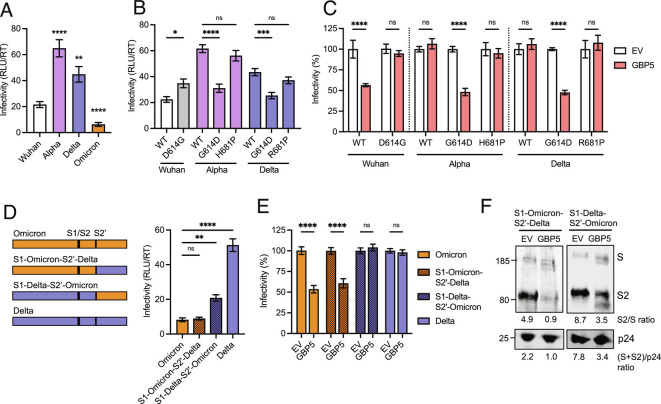
Mapping spike determinants mediating the differential sensitivity of SARS-CoV-2 isolates to GBP5 inhibition. (*A*) Comparison of particle infectivity (RLU/RT) of spike PVs on Caco2 cells in the absence of GBPs. (*B* and *C*) Wuhan D614G, Alpha G614D, and H681P, and Delta G614D and R681P spike mutants were generated and tested for PV infectivity and GBP5 sensitivity. (*B*) Comparison of spike mutants PV particle infectivity (RLU/RT) on Caco2 cells in the absence of GBP expression. (*C*) Percentage infectivity of spike mutants PV made in the presence of GBP5 normalized to empty vector (EV) control for each spike (set to 100%). (*D*–*F*) Chimeric spikes were generated with S2’ domains swapped between Delta and Omicron spikes to produce S1-Omicron-S2’-Delta and S1-Delta-S2’-Omicron chimeras (schematic). Indicated spike PV were produced in the presence of GBP5 or EV control and titrated on Caco2 cells. (*D*) Raw infectivity (RLU/RT) values in the absence of GBP5 expression (EV control) and (*E*) percent infectivity normalized to EV control are shown. (*F*) S1-Omicron-S2’-Delta and S1-Delta-S2’-Omicron spike PVs made in the presence of GBP5 or EV control were immunoblotted for spike and p24. Bars show mean ± SEM from three independent experiments. One-way ANOVA (*A*, *D*) or two-way ANOVA (*B*, *C*, *E*) with Dunnett’s posttest were used. ns, not significant; *P < 0.05; **P < 0.01; ***P < 0.001; ****P < 0.0001.

The D614G mutation in spike manifested as an early host adaptation in Wuhan-like isolates that enhances infectivity and is now ubiquitous in circulating SARS-CoV-2 variants (31–33). Alpha and Delta contain the infectivity-enhancing D614G mutation in spike (*SI Appendix*, Fig. S1*B*), whereas Wuhan-Hu-1 and VIC do not. Having observed that the most infectious PV (Alpha and Delta) were resistant to GBP restriction, whereas the less infectious Wuhan-Hu-1 PV was sensitive (Fig. 3*A*), we sought to test whether the D614G substitution may explain the resistance of Alpha and Delta VOCs to GBP2/5 inhibition of infectivity. Strikingly, introducing the D614G mutation into Wuhan-Hu-1 spike (Wuhan D614G) completely rescued PV from GBP inhibition (Fig. 3*C*), although spike cleavage was still inhibited (Fig. 2 *A* and *B*). Consistent with previous reports, D614G also rendered Wuhan-Hu-1 PV more infectious on a per particle basis, but it remained less infectious than Alpha and Delta (Fig. 3*B*) (31–33). Importantly, reverting Alpha and Delta spikes back to the ancestral Wuhan-Hu-1 by introducing a G614D substitution resulted in these PV becoming sensitive to GBP restriction, evidenced by a significant 50% reduction in infectivity (Fig. 3*C*). Consistent with the optimized FCS not explaining Alpha and Delta resistance to GBPs, reverting the FCS mutations in Alpha (H681) and Delta (R681) spike back to Wuhan-Hu-1 like sequences (creating Alpha H681P and Delta R681P, respectively) had no effect on the restriction phenotype, with these mutants remaining fully resistant to GBP-mediated inhibition of infectivity (Fig. 3*C*). Importantly, increasing the amount of spike incorporated into Wuhan-Hu-1 PV using a construct in which the 19 residues of the spike cytoplasmic tail are deleted (ΔCT) boosted spike incorporation and particle infectivity as described previously (34, 35), but did not rescue from GBP restriction (*SI Appendix*, Fig. S6 *A*–*C*). Similarly, titrating increasing amounts of full-length Wuhan-Hu-1 spike plasmid into 293T cells during PV production in the presence of GBP also failed to rescue from GBP restriction (*SI Appendix*, Fig. S6*G*). Extending this, reducing the amount of Alpha and Delta spikes transfected into 293T cells failed to sensitize these PV to GBP restriction (*SI Appendix*, Fig. S6 *J* and *K*). Taken together, these data identify the D614G substitutions as the evolutionary change allowing Alpha and Delta to evade GBP-mediated restriction.

### The S1 Domain of Spike Confers Sensitivity of Omicron to GBP Restriction.

Omicron also contains the same D614G substitution as Alpha and Delta but remained sensitive to GBPs, behaving like Wuhan-Hu-1 (Fig. 3*A*). However, in addition to D614G, Omicron contains a large number of antibody escape mutations in its spike compared with other SARS-CoV-2 isolates (*SI Appendix*, Fig. S1*B*) (36–39), suggesting that this constellation of changes may have negatively influenced spike activity and its capacity to escape GBP restriction. Concordantly, Omicron PV infectivity was significantly lower than other variants tested (Fig. 3*A*), consistent with reports that Omicron displays reduced infectivity in cell lines commonly used to study SARS-CoV-2 entry (36–39) and in animal models (38). These spike changes are present both in the receptor-binding S1 subunit, and in the S2’ domain that harbors the viral fusion peptide (*SI Appendix*, Fig. S1*B*). Therefore, we generated chimeric Omicron–Delta spikes to determine whether S1 or S2’ determines Omicron sensitivity to GBPs (Fig. 3*D*). We used Delta because its spike has the highest fusogenicity (40) and is resistant to GBPs. Fig. 3 *D*–*F* shows that replacing the S1 domain of Omicron with that of Delta (creating S1-Delta-S2’-Omicron spike) conferred complete resistance to GBP inhibition of infectivity. By contrast the Delta-S2’ domain did not rescue, and this spike (S1-Omicron-S2’-Delta) remained GBP sensitive, phenocopying the native Omicron spike. Similar to both the full-length Delta and Omicron spikes, these chimeric spikes remained sensitive to GBP inhibition of furin-cleavage (Fig. 3*F*). Finally, increasing Omicron spike incorporation into PV using the ΔCT spike mutant, or titrating increasing amounts of full-length Omicron spike plasmid into 293T cells during PV production failed to rescue Omicron from GBP restriction (*SI Appendix*, Fig. S6 *D*–*F* and *H*), similar to Wuhan-Hu-1. These data suggest that restriction of Wuhan-Hu-1 and Omicron by GBPs cannot be simply explained by reduced spike incorporation into virions. We conclude that the S1 domain contains the determinant for Omicron’s sensitivity to GBP-mediated inhibition of infection, and that for Omicron the changes within the S1 domain of spike have compromised the ability of the D614G mutation to confer resistance to GBPs.

### GBPs Do Not Sensitize to Restriction by Endosomal IFITM2 and 3.

**Fig. 4.**
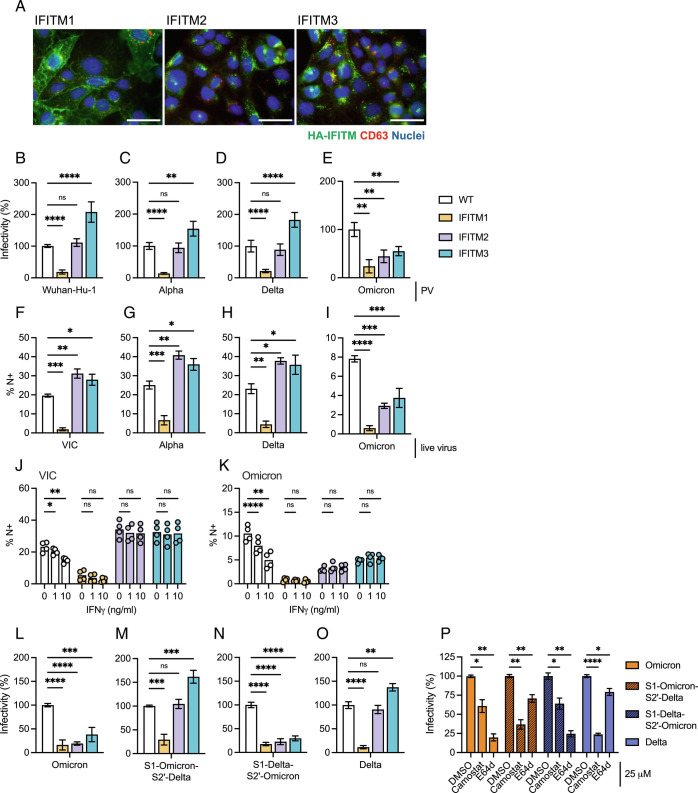
Omicron is uniquely restricted by IFITM2/3, which maps to the spike S2’ domain. (*A*) Localization of HA-tagged IFITMs 1-3 in Caco2 cells was assessed by immunofluorescence imaging. HA-tag IFITM (green), CD63 (red), and nuclei (blue). (Scale bar is 50 μm.) (*B*–*E*) Infection of IFITM-expressing Caco2 cells with (*B*) Wuhan-Hu-1, (*C*) Alpha, (*D*) Delta, and (*E*) Omicron spike PV. Percent infectivity normalized to WT Caco2 cells (no IFITM over-expression) are shown. (*F*–*I*) IFITM-expressing Caco2 cells were infected with SARS-CoV-2. Shown is percent N+ cells at 24 hpi of (*F*) VIC, (*G*) Alpha, (*H*) Delta, and (*I*) Omicron isolates. Mean ± SEM from three independent experiments are shown (*J*, *K*) Calu-3 cells were treated with indicated doses of IFNγ for 8 h to induce expression of GBP2/5 and infected with indicated SARS-CoV-2 variants as shown in Fig. 1. At 36 hpi, virus-containing supernatant of (*J*) VIC and (*K*) Omicron isolates were harvested, and equal doses of virus (E copies/cell) from the supernatants were used to infect WT or IFITM expressing Caco2 cells for 24 h. Infection was quantified as the percentage N+ cells. Mean and individual replicates from two independent experiments are shown. (*L*–*O*) IFITM-expressing Caco2 cells were infected with (*L*) Omicron, (*M*) S1-Omicron-S2’-Delta, (*N*) S1-Delta-S2’-Omicron, and (*O*) Delta spike PV. Percent infectivity normalized to WT Caco2 cells (no IFITM overexpression) are shown. (*P*) Indicated spike PV were used to infect Caco2 cells pretreated with 25 μM Camostat or E64d. Shown is percent infection normalized to DMSO control. One-way ANOVA (*B*–*I*, *L*–*O*) or two-way ANOVA (*J*, *K*, *P*) with Dunnett’s posttest was used. ns, not significant; *P < 0.05; **P < 0.01; ***P < 0.001; ****P < 0.0001.

It has been reported that mutating the FCS in Alpha to interfere with furin-cleavage can modulate sensitivity to inhibition by endosomal IFITM2 (12, 22). Having shown that GBPs interfere with SARS-CoV-2 spike cleavage, we sought to test whether this may sensitize SARS-CoV-2 to IFITM-mediated inhibition. IFITMs are another family of spike-targeting interferon-inducible restriction factors that can inhibit infection of a range of viruses, including SARS-CoV-2 (7, 12, 21–24, 41), by perturbing viral fusion with host cell membranes and thus inhibiting infection of target cells. IFITMs are differentially localized in cells with IFITM1 being found mostly at the plasma membrane and IFITM2/3 predominantly endosomal. To explore the effects of GBPs on IFITM sensitivity, we used Caco2 cells stably overexpressing either IFITM1, 2, or 3 and confirmed the expected IFITM localization by immunofluorescence microscopy (Fig. 4*A*). We first established the IFITM restriction phenotype in these cells. Consistent with TMPRSS2-dependent plasma membrane fusion, IFITM1 was found to potently and significantly inhibit infection of early-lineage viruses (Wuhan-Hu-1 and VIC) and VOCs Alpha and Delta in both PV (Fig. 4 *B*–*D*) and live virus infections (Fig. 4 *F*–*H*). By contrast, IFITM2 and IFITM3 did not inhibit but rather enhanced infection in agreement with previous studies (12, 22, 24, 41, 42). Notably, Omicron was unique among SARS-CoV-2 viruses in being sensitive to inhibition by endosomal IFITM2 and 3 in Caco2 cells (Fig. 4 *E* and *I*). This is consistent with Omicron favoring TMPRSS2-independent endosomal entry pathways, evidenced by increased sensitivity to the cathepsin inhibitor E64d and reduced sensitivity to the TMPRSS2 inhibitor Camostat (*SI Appendix*, Fig. S7), in agreement with others (36–39, 43). Infecting IFITM-expressing Caco2 cells with VIC or Omicron (sensitive to GBP restriction) produced from IFNγ-treated Calu-3 (from Fig. 1) showed no difference in the sensitivity of these viruses to IFITMs when compared with virus produced from untreated Calu-3 cells (Fig. 4 *J* and *K*). Similar results were obtained using Wuhan-Hu-1 and Omicron PV made in the presence of overexpressed GBP5, where we found no change in IFITM restriction and no increase in inhibition by endosomal IFITM2 or 3 (*SI Appendix*, Fig. S8). Together, these data show that inhibiting furin cleavage by GBPs does not make a virus that is resistant to IFITMs become sensitive to restriction.

Finally, to map the domains in Omicron that dictate its sensitivity to endosomal IFITM2 and 3, we used our chimeric Omicron–Delta spike mutants (Fig. 3*D*). Fig. 4 *L*–*O* show that replacing the S2’ domain of Omicron with that of Delta rendered this PV resistant to IFITM2 and 3 inhibition and sensitive to IFITM1, thus behaving like Delta. This S1-Omicron-S2’-Delta spike PV also became sensitive to the TMPRSS2 inhibitor Camostat and resistant to the endosomal E64d inhibitor, thus behaving like Delta (Fig. 4*P*), a finding supported by recent studies (37, 39). These results identify the S2’ domain of spike as the determinant for IFITM sensitivity, by contrast to GBP-sensitivity that is mediated by the S1 domain, and reveal that Omicron’s unique sensitivity to IFITM2 and 3 (when compared with other SARS-CoV-2 variants) is dictated by its altered entry route.

## Discussion

Innate immunity is a potent first-line host cell defense against viruses, up-regulating a group of ISGs which can act directly as restriction factors, targeting key steps in viral replication and collectively inducing an antiviral state. SARS-CoV-2 triggers innate immune sensing and induces an interferon-response (11, 25, 44, 45), upregulating canonical ISGs including GBP2 and 5 in primary human airway epithelial cells (20). Evolution of mutations outside of spike allow for SARS-CoV-2 evasion/antagonism of innate immune sensing (11); however, spike itself is a target of the innate immune response.

Here we report that the interferon-inducible restriction factors GBP2 and 5 interfere with SARS-CoV-2 spike cleavage, and significantly inhibit infection by the early-lineage SARS-CoV-2 strains Wuhan-Hu-1 and VIC, but that previously dominant VOCs Alpha and Delta have evolved to evade GBP-mediated inhibition of infectivity. Notably, Alpha and Delta have both evolved an optimized FCS by acquiring the P681H and P681R substitutions, respectively, resulting in enhanced spike processing (12, 14, 15); however, we find that evolution of these VOCs to escape GBP restriction is not due to this optimized FCS. Instead, we find that it is the presence of the now ubiquitous D614G substitution in spike that mediates Alpha and Delta resistance to GBPs. Specifically, we show that reverting Alpha and Delta to 614D led these PV becoming sensitive to GBP-mediated inhibition of infection, behaving like Wuhan-Hu-1 and VIC. By contrast, introducing the 614G mutation into Wuhan-Hu-1 spike rescued from GBP inhibition and increased spike infectivity.

The D614G variant has been reported to enhance SARS-CoV-2 spike infectivity (31, 33, 46, 47) by stabilizing the S1/S2 subunit noncovalent association and altering spike conformation, shifting it toward a more open, fusion competent state, without increasing ACE2-binding affinity (33, 46, 48). Therefore, by allowing spike to adopt the more open conformation that is on pathway for fusion, the 614G change essentially makes spike better primed for function. Consistent with this, we saw a correlation between the presence of 614G in spike and increased PV infectivity. By contrast, we found that early lineage isolates that do not contain D614G cannot overcome the restriction by GBP. The fact that we could not rescue Wuhan-Hu-1 or Omicron from GBP restriction by increasing spike incorporation argues against the effects of GBPs being mediated simply by reducing overall spike content in virions. Instead, our data suggest that GBPs restrict SARS-CoV-2 infectivity by impairing furin-cleavage of spike and altering spike processing (glycosylation), leading to reduced spike function that consequently reduces particle infectivity and entry into target cells. However, evolution for the D614G substitution has allowed Alpha and Delta to overcome the loss of spike function caused by GBPs by improving spike activity and particle infectivity, allowing these VOCs to evade restriction. It is therefore tempting to speculate that the selection for and subsequent dominance of D614G in SARS-CoV-2 isolates was due, in part, to adaptation to host in order to escape from interferon-induced innate immunity, as well as inherent effects of D614G on improving spike function to increase transmissibility.

It has been shown that interfering with spike cleavage by mutating the FCS in Alpha sensitizes virus to inhibition by another spike targeting restriction factor, namely endosomal IFITM2 (12, 22). However, despite GBPs perturbing spike cleavage and reducing Wuhan-Hu-1 infectivity, we did not find that GBPs sensitized SARS-CoV-2 to inhibition by endosomal IFITM2 or 3. We explain this by GBPs reducing, but not completely preventing, spike S1/S2 cleavage, thus allowing Wuhan-Hu-1 to retain a preference for plasma membrane entry, and therefore retaining sensitivity to plasma membrane localized IFITM1. These results are consistent with reports that other cellular proteases can mediate some spike processing at the polybasic cleavage site in the absence of furin (8). It is notable however, that although GBPs did not sensitize to IFITMs, combining GBP5 in producer cells with IFITM1 in target cells led to an almost complete inhibition of Wuhan-Hu-1 infection when compared with infection in the absence of these restriction factors. Thus, during an innate immune response to viral infection where multiple ISGs including GBP2/5 and IFITM1/2/3 are induced, we might expect to see stronger inhibitory effects.

We show that Omicron is unique among VOCs we tested in being sensitive to inhibition by GBP2/5, despite containing the same D614G substitution as Alpha and Delta. Using chimeric Omicron–Delta spike mutants, we mapped the determinant of Omicron’s sensitivity to GBPs to the S1 domain. We propose that the constellation of mutations present in Omicron spike compared with other VOCs has compromised the ability of D614G to overcome the inhibitory effects of GBPs. Consistent with this notion, Omicron was significantly less infectious than other SARS-CoV-2 isolates we tested, indicative of the multiple mutations impacting spike function. Given the significant number of substitutions in Omicron, this is not surprising. Further work will be needed to define precisely which combination of changes in Omicron S1 mediates its sensitivity to GBP2/5 and compromise the effects of D614G in driving escape. Omicron was also unique in being sensitive to inhibition by IFITM1, 2, and 3, unlike other isolates we tested that were not inhibited by IFITM2 and 3. This is explained by Omicron having evolved toward an altered entry route of TMPRSS2-independence and endosomal-dependent fusion (36, 37, 39), thus exposing it to endosomal IFITMs. The domains that mediate Omicron’s sensitivity to IFITM2 and 3 are distinct from those that mediate GBP-sensitivity, and we show that it is the S2’ domain of spike that dictates its entry phenotype and sensitivity to endosomal IFITM2 and 3. Specifically, replacing the S2’ domain in Omicron spike with that of Delta (S1-Omicron-S2’-Delta), converted the phenotype rendering this PV “Delta-like” and resistant to IFITM2/3 by switching Omicron PV entry back to TMPRSS2-dependence and away from endosomal entry. While this manuscript was in preparation, Omicron subvariants BA.4 and BA.5 began dominating infections globally. We also confirmed that like BA.1, BA.2 and BA.4/5 spikes are sensitive to both GBP and IFITM restriction and retain the same preference for TMPRSS2-independent entry (*SI Appendix*, Fig. S9). The in vivo interplay between Omicron and innate immunity, restriction factors, and tropism remains ill-defined. It is possible that the expression of different innate immune restriction factors varies across cells/tissues, such that Omicron has evolved into a niche that allows it to avoid or tolerate GBPs and IFITMs. Moreover, IFITM2 and 3 are suggested to act as cofactors for SARS-CoV-2 in some cases (41, 42), and we and others also see enhancement of early-lineage isolates and Alpha and Delta infection by IFITM2/3 (Fig. 4) (12, 22, 24, 41, 42), therefore it cannot be excluded that Omicron may similarly exploit IFITMs in some settings. It is clear that SARS-CoV-2 needs to balance efficient cell entry with evasion of compartmentalized restriction factors, and it will be intriguing to see how Omicron does this to successfully infect target cells in vivo. Omicron has evidently evolved to do things differently, but effectively, and exploit a different cellular niche, one to which it is clearly well adapted.

SARS-CoV-2 VOCs have evolved separately from early lineage strains and not from each other. It is therefore not surprising that these VOCs have explored different evolutionary solutions to the problems they faced, and that the selective pressures encountered by each VOC are not identical. For example, Alpha and Delta evolved prior to significant levels of adaptive immunity in the population. By contrast Omicron, the first real antibody escape variant, evolved at a time of much greater population level humoral immunity, therefore Omicron has been exposed to different selective pressures, requiring different evolutionary solutions. Our data showing differential sensitivity of Alpha/Delta vs Omicron to GBP and IFITM restriction, as well as others showing significant Omicron antibody escape (36, 39), are consistent with this, reinforcing the notion that Omicron has taken a different evolutionary path to preceding VOCs. We propose a scenario in which evolution of Omicron spike for neutralizing antibody escape has influenced the ability to evade innate immunity. The critical balance between viral evasion of innate and adaptive immunity has precedent. This is borne out of studies of HIV-1 evolution in a host, where HIV-1 isolates from early in infection (so called transmitter/founder viruses) are completely resistant to IFITM restriction, but overtime, the selective pressure from adaptive immunity, and the resulting neutralizing antibody escape mutations in HIV-1 Env, leads to viral isolates having increased sensitivity to IFITMs and interferons (49). We propose that similar processes have occurred during SARS-CoV-2 evolution to host, in which the need to escape from neutralizing antibody became the dominant selective pressure on Omicron, resulting in a compensatory, but tolerable, increase in sensitivity to innate immunity, while also impacting on spike activity and cell tropism. We predict that this interplay between evasion of innate and adaptive immunity, and the consequences for transmission and tropism, will be features of future SARS-CoV-2 evolution, and emergence of new VOCs, and that linking this evolution to phenotype will become important aspects for understanding and predicting SARS-CoV-2 biology, and ultimately pathogenesis.

## Materials and Methods

### Cells.

HEK293T/17 cells (abbreviated herein as 293T cells) were obtained from American Type Culture Collection (ATCC, CRL-11268). Caco2 cells were a gift from Dalan Bailey (Pirbright Institute) and originally obtained from ATCC. Calu-3 cells were purchased from AddexBio (C0016001). Vero.E6 cells were obtained from the National Institute for Biological Standards and Control. HeLa-TZM-bl cells (expressing luciferase and beta-galactosidase under the control of HIV-1 LTR) were obtained from the Centre for AIDS Reagents (CFAR). All cell lines were grown in Dulbecco’s modified Eagle’s medium (DMEM, Thermo Fisher Scientific) supplemented with 10% fetal bovine serum (Labtech) and 1% Pen Strep (penicillin-streptomycin, Thermo Fisher Scientific), and maintained in humidified 5% CO2 incubators at 37 °C. Cells were passaged every 2 to 4 d when they reached 80 to 90% confluency. Caco2 cells were transduced with IFITM1/2/3 lentivectors as described previously (50) and selected with 10 μg/mL puromycin (Merck) to produce stable cell lines expressing individual HA-tagged IFITM proteins. IFITM expression was confirmed by flow cytometry. Primary normal (healthy) bronchial epithelial (NHBE-A) cells were cultured for 5 to 7 passages and differentiated at an air-liquid interface as previously described (11). After 21 to 24 d of differentiation, cells were used in infection experiments.

### Plasmids.

SARS-CoV-2 spike expression vectors were originally synthesized by Genewiz and subcloned into pcDNA3.1+ vector. All spike sequences are full-length, unless otherwise stated. Wuhan-Hu-1 WT (51), Wuhan-Hu-1 D614G, and Alpha (52) spike expression vectors were gifts from Laura McCoy (UCL). Delta and Omicron BA.1 spike expression vectors were a gift from Katie Doores (King’s College London). Wuhan-Hu-1 WT, Wuhan-Hu-1 ΔFCS, Omicron BA.1, BA.2, and BA.4/5 spike ΔCT expression vectors were a gift from Wendy Barclay (Imperial College London) (7, 37). SARS-CoV-1 spike and MERS-CoV spike expression vectors were a gift from Joe Grove (Centre for Virus Research, Glasgow). Plasmid encoding HIV-1 Env pSVIII_JRFL was a gift from Laura McCoy (UCL). Plasmid encoding full-length HIV-1 pNL4.3 was donated by Dr M Martin and obtained from CFAR. Lentiviral backbone packaging plasmid (expressing HIV Gag, Pol, Tat and Rev) p8.91 and the reporter plasmid-encoding luciferase gene pCSLW were a gift from Greg Towers (UCL). GBP expression vectors encoding HA-tagged GBPs (GBP2 WT, GBP2 C588A, GBP5 WT, GBP5 C583A) and BFP reporter expressed from an IRES (17, 18) were a gift from Daniel Sauter (University Hospital Tubingen).

### Mutagenesis.

Mutagenesis was performed as described in *SI Appendix, Methods*.

### SARS-CoV-2 Viruses.

SARS-CoV-2 isolates VIC (BetaCoV/Australia/VIC01/2020, lineage B), IC19 (hCoV-19/England/IC19/2020, lineage B.1.13) and Alpha (hCoV-19/England/204690005/2020, lineage B.1.1.7) have been described previously (11). SARS-CoV-2 Delta (lineage B.1.617.2) and Omicron (lineage B.1.1.529/BA.1) isolates were a kind gift from Wendy Barclay (Imperial College London, UK) (14, 37). Viruses were propagated by infecting Caco2 cells at MOI 0.01 TCID50 per cell, in DMEM culture medium supplemented with 1% FBS and 1% penicillin/streptomycin, at 37 °C. Virus was collected at 72 hpi and clarified by centrifugation at 2,100 × g for 15 min at 4 °C to remove any cellular debris. Virus stocks were aliquoted and stored at −80 °C. Virus stocks were quantified by extracting RNA from 100 µL supernatant with 1 µg carrier RNA using Qiagen RNeasy clean-up RNA protocol, before measuring viral E RNA copies per mL by RT-qPCR as described previously (25).

### Live Virus Infections.

Caco2 (1 × 10^5^ cells/well) were seeded in 24-well plates one day before infection. Cells were infected with 1000 E RNA copies per cell in 200μL culture medium. After 2h incubation at 37 °C, cells were carefully washed with PBS to remove excess virus, and a fresh culture medium was added. For inhibition assays, cells were pretreated with inhibitors at the indicated concentrations for 2 h prior to infections and maintained throughout the experiment. At the indicated time points, cells were collected for analysis. For Calu-3 cell infections, 2 × 10^5^ cells/well were seeded into 12-well plates and grown until confluent. Where indicated, cells were pretreated with indicated concentrations of recombinant human IFNγ (Peprotech) for 8 h before cells were infected with 1000 E RNA copies SARS-CoV-2 per cell in 400 μL culture medium. The inoculum was thoroughly washed off with PBS after 2 h, and a fresh culture medium added. At 36 hpi, cells were harvested for protein lysates and flow cytometry. Culture supernatants from infected cells were clarified by centrifugation at 2,100 × g for 15 min at 4 °C and viral E RNA copies measured by RT-qPCR. To determine infectivity of these viral supernatants, Caco2 cells were pretreated with 5 μM Ruxolitinib (Bio-Techne) for 1 h to inhibit JAK signaling (from carryover interferon) and then infected with 1000 E copies/cell of virus as described above and harvested at 24 hpi for flow cytometry analysis. Ruxolitinib was maintained throughout. Primary human airway epithelial cells (HAEs) were infected by adding 1500 E copies/cell to the apical side for 3 h at 37 °C. Supernatant was then removed and cells were gently washed twice with PBS. All liquid was removed from the apical side, and the basal medium was replaced with fresh Pneumacult ALI medium for the duration of the experiment. For IFNγ-stimulation, 10 ng/mL recombinant IFNγ (Peprotech) was added basally 12 h before HAE infection and maintained throughout the experiment. Intracellular replication was determined at 72 hpi, and viral release was measured at 24 and 72 h by extracting viral RNA from apical PBS washes as described previously (11).

### *GBP2/5* RNAi Depletion.

Calu-3 cells (2 × 10^5^ cells/well) were seeded into 12-well plates and transfected with 20 pmol siRNA SMART pool against *GBP2* (L-011867-00-0005) and GBP5 (L-018178-00-0005) or nontargeting control (D-001810-10-05) (Dharmacon) using Lipofectamine RNAiMAX Transfection Reagent (Invitrogen). Cells were treated with siRNA transfection reagents at both 120 h and 36 h before infection. Cells were then treated with indicated concentrations of recombinant human IFNγ (Peprotech) for 8 h before infection as described above. GBP2/5 depletion was confirmed by immunoblotting (described below).

### Pseudovirus Production.

Spike pseudoviruses (PVs) were made by cotransfection of spike, p8.91 and pCSLW plasmids as described previously (53). To determine GBP inhibition, PV plasmids were transfected together with GBP vector or empty vector (EV) control expressing only BFP reporter. Briefly, 5 × 10^4^ 293T cells were seeded onto 24-well plates for 24 h and then transfected with 260 ng p8.91, 260 ng pCSLW, 40 ng spike, and 20 to 120 ng GBP vectors or EV control using Fugene6 (Promega). For larger scale production cells were seeded in 6-well plates with cell numbers and transfection reagents scaled up fivefold. For HIV-1 Env PV, 293T cells were seeded as described above and transfected with 240 ng p8.91, 240 ng pCSLW,120 ng pSVIII-JRFL Env, and indicated doses of GBP or empty vector control.

PV supernatants were collected at 48 and 72 h posttransfection and purified through 0.45 μm centrifuge tube filters (Corning) or 0.45 μm syringe-filters (Starlab) and used within 24 h without freeze–thawing. The amount of PV in the supernatant was determined by measuring the supernatant RT activity using SYBR-green-based product enhanced reverse transcription assay (SG-PERT) by qPCR, performed as described previously (54).

### Pseudovirus Infection.

Target cells were seeded into white 96-well plates 24 h before infection (Caco2 and Vero.E6 cells seeded at 1.5 × 10^4^ cells/well, and HeLa-TZMbl cells seeded at 1 × 10^4^ cells/well). Cells were infected with increasing doses (2 to 15 mU RT/well) of PV supernatant (to confirm linear increase in infection at increased PV doses) and incubated at 37 °C for 48 h without changing the medium. Luciferase expression (RLU) was measured at 48 h postinfection using BrightGlo substrate (Promega) according to the manufacturer’s instruction on the Glomax luminometer (Promega). For inhibitor studies, cells were pretreated before infections for 2 h with Camostat mesylate (Apexbio, 0.2 to 100 μM) or E64d (Focus Biomolecules, 0.2 to 25 μM). To obtain infectivity (RLU/RT) values, RLU values were normalized to input supernatant RT activity (measured by SG-PERT assay, described above).

### GBP Inhibition Assay.

Spike PV, HIV-1 Env PV, or HIV-1 virus were made in the presence of increasing doses of GBP2/5 or their mutants or empty vector control as described above. Supernatant RT activity (RT units) was measured by SG–PERT assay to determine PV or virus content in supernatants. As indicated, different cell lines were infected with equal doses of PV/virus supernatants for 48 h and luciferase expression (RLU) was measured as describe above. Infectivity of the supernatant was determined as a ratio of RLU to RT units (RLU/RT) and normalized to empty vector control (no GBP), set at 100% for each PV or virus.

### IFITM Inhibition Assay.

Caco2 cells, WT or stably expressing IFITM1/2/3 (described above), were infected with indicated spike PVs, and luciferase expression was measured 48 h postinfection as described above. Infectivity (RLU/RT) was normalized to Caco2 WT control (100%). Alternatively, spike PVs were made in the presence of GBP5 or EV control and used to infect Caco2 WT- and IFITM-expressing cells. Infection of Caco2-IFITM1/2/3 cells with live SARS-CoV-2 virus was done as described above.

### HIV-1 Infection.

HIV-1 infections were performed as described in *SI Appendix, Methods*.

### Quantitative PCR.

Quantitative PCR was performed as described in *SI Appendix, Methods*.

### Immunoblotting.

Immunoblotting was performed as described in *SI Appendix, Methods*.

### Flow Cytometry.

Flow cytometry was performed as described in *SI Appendix, Methods*.

### Immunofluorescence Microscopy.

Immunofluorescence microscopy was performed as described in *SI Appendix, Methods*.

### Statistical Analysis.

Statistical significance was calculated using Prism 9 (GraphPad Prism) using indicated statistical tests and significance was assumed when *P* < 0.05.

## Supplementary Material

Appendix 01 (PDF)

## Data Availability

All study data are included in the article and/or *SI Appendix*.
