# Supplementary material for: SARS-CoV-2 evolution influences GBP and IFITM sensitivity
Source: Proc Natl Acad Sci U S A. 2023 Jan 24;120(5):e2212577120. doi: 10.1073/pnas.2212577120 (PMC9945951; doi:10.1073/pnas.2212577120)
Supplement: Supplementary file 1 — Appendix 01 (PDF) [file pnas.2212577120.sapp.pdf]

PNAS

Supporting Information for

SARS-CoV-2 evolution influences GBP and IFITM sensitivity

Dejan Mesner, Ann-Kathrin Reuschl, Matthew V.X Whelan, Taylor Bronzovich, Tafhima Haider, Lucy G. Thorne, Roberta Ragazzini, Paola Bonfanti, Greg J. Towers and Clare Jolly

Clare Jolly
Email: c.jolly@ucl.ac.uk

This PDF file includes:

Supporting text including methods
Figures S1 to S9
SI References

Supporting Information Text

Supplementary Methods:

Mutagenesis

Wuhan-Hu-1 ΔFCS spike ΔCT vector was converted to full-length by mutating stop codon at position 1255 into lysine residue (*1255K) as found in WT sequence. Mutagenesis was performed using QuikChange Lightning site-directed mutagenesis kit (Agilent) and following primers: 5'-GTCCTCGTCGAACTTGCAGCAGCTGCCAC-3' and 5'-GTGGCAGCTGCTGCAAGTTCGACGAGGAC-3'.

Delta-Omicron chimeras were constructed by Gibson assembly cloning method. Delta and Omicron BA.1 S2' domain sequence were PCR amplified using following primers:

5'-GCAGTATGGCGATTGTCTGGGCGAC-3'

5'-CGTACACGGTATTGTTCACAATGCCGATCAC-3'.

Delta and Omicron BA.1 vector sequence excluding S2' domain were amplified using following primers:

5'-GTGATCGGCATTGTGAACAATACCGTGTACG-3'

5'-GTCGCCCAGACAATCGCCATACTGC-3'

The S1 and S2' domain fragments were assembled using NEBuilder HiFi DNA Assembly Cloning Kit (New England Biolabs) according to manufacturer's instructions. All mutagenesis and cloning was confirmed by sequencing.

HIV-1 infection

293T cells were seeded in 24-well plates as described above and transfected with 120ng pNL4.3 and indicated doses of GBP or empty vector control using Fugene6 to produce infectious HIV-1 virus in presence or absence of GBPs. Virus-containing supernatant was harvested and RT activity measured as described above. HeLa-TZMbl were seeded into white 96-well plates 24 h before infection ($1x10^4$ cells/well) and infected with 2-10 mU RT of virus supernatant/well. Luciferase activity was measured 48 h post-infection as described above.

qRT-PCR

GBP2 and *GBP5* gene expression in IFN-gamma treated human airway epithelial cells (HAEs) was measured by qRT-PCR as described previously (1). Gene expression was determined using the $2^{-\Delta\Delta Ct}$ method and normalised to *GAPDH* expression. The following

primers were used: *GAPDH* fw: ACATCGCTCAGACACCATG, rv: TGTAGTTGAGGTCAATGAAGGG;
GBP2 fw: CTATCTGCAATTACGCAGCCT, rv: TGTTCTGGCTTCTTGGGATGA;
GBP5 fw: CCATGTGCCTCATCGAGAACT, rv: ACAGGTTGCGTAATGGCGAC.

Immunoblotting PV and live viruses were concentrated and purified from the supernatant by centrifugation (2h 13,000g, 4^0C) through a 20% sucrose cushion and resuspended in 20 µl Leammli buffer. Cells were harvested, lysed in RIPA buffer and protein concentration determined by the BCA assay (Thermo Fisher Scientific). Twenty micrograms of cell lysate and an equal volume of purified virus were separated by SDS-PAGE, transferred to nitrocellulose membrane and membranes probed using the following primary antibodies: anti-SARS-CoV Spike (100% overlap with the SARS-CoV-2 epitope)(Invitrogen, PA1-41165, 1:1000 or GeneTex 1A9, 1:1000) anti SARS-CoV nucleocapsid (N) protein monoclonal antibody (clone CR3009, gift from Laura McCoy, 0.5 µg/ml), anti-HA-tag (Biolegend, 16B12, 1:2000), anti-Gag p24 (CFAR, ARP432, 1:2000), anti-GBP5 (Cell Signaling, D3A5O, 1:1000), anti-GBP2 (Santa Cruz, G-9, 1:200), and anti-tubulin (Sigma, DM1A, 1:1000). Primary antibodies were incubated overnight at 4°C with agitation and detected with fluorescent secondary antibodies: anti-Rabbit IgG (IRDye 800CW, Abcam, ab216773, 1:10,000), anti-Mouse IgG (IRDye 680RD, Abcam, ab216776, 1:10,000) and anti-Human IgG (IRDye 800CW, Licor, 925-68078, 1:10,000), and imaged with an Odyssey Cxl Infrared Imager (Licor). Immunoblots were analysed with Image Studio Lite software. PNGase F treatment to remove N-linked glycosylation was performed by treating 20 µg cell lysates with 1 µl PNGase F (New England Biolabs) according to manufacturer's instructions and analysed by immunoblotting as described above.

Immunofluorescence Microscopy
Caco2 cells or 293T cells were fixed in 4%PFA (Sigma) for 15 minutes, followed by permeabilisation in 0.25%Triton-TX100 (Sigma) for 15 minutes. A blocking step was carried out for 1h at room temperature with 1%BSA (Sigma) and 0.1% Triton TX100 in PBS. Primary antibody incubation was carried out for 1h at RT with human anti-SARS-CoV-2 Spike, (clone CR3022, gift from Laura McCoy, UCL) rabbit anti-Calnexin (Abcam, ab22595) to label the Endoplasmic Reticulum (ER), mouse anti-HA (clone HA-7, H6908, Sigma-Aldrich; and clone 16B12, 901523, Biolegend) to label HA-IFITM1/23 and HA-

GBP5, and mouse anti-CD63 (IB5, a gift from M. Marsh, UCL) to label endogenous CD63. Primary antibodies were detected with secondary anti-human AlexaFluor-488, anti-rabbit AlexaFluor-488, anti-rabbit AlexaFluor-568, anti-mouse AlexaFluor-568 and anti-mouse AlexaFluor-647 conjugates (Jackson Immuno Research) for 1h. All cells were labelled with Hoechst33342 (H3570, Thermo Fisher). Images were acquired using the WiScan® Hermes 7-Colour High-Content Imaging System (IDEA Bio-Medical, Rehovot, Israel) at magnification 40X/0.75NA or with a VT-iSIM structured illumination microscope (VisiTech, Sunderland, UK) at 60X/1.4 NA oil with 1.5X Zoom. For Hermes imaging, 3-colour automated acquisition was carried out sequentially. For VT-iSIM imaging, 4-colour sequential scanning of the medial plane was carried out. Images were deconvolved using NIS-Elements (Nikon, Tokyo, Japan) using a Richardson-Lucy method with 5 iterations. A 100-pixel rolling ball background subtraction was carried out for visualisation. Scale bars and RGB composite images were created in in FIJI ImageJ software package (2).

Image Analysis

Colocalisation analysis of Spike with ER-marker Calnexin and GBP5 was carried out for EV (Empty Vector +Spike) or +GBP5 (GBP5 and Spike) expressing 293T cells using Fiji ImageJ. Briefly, spike positive cells were manually identified and confirmed for GBP5 and BFP expression. Regions of Interest (ROI) were manually drawn, and a bounding box crop was applied. A segmentation mask was generated by auto-thresholding of the spike channel within the cell and manual subtraction of extracellular signal from all channels within the ROI. Pearson’s correlation coefficient was quantified for spike and Calnexin, and spike and GBP5 using the Coloc2 plugin. For each cell, colocalisation analysis was restricted to within the borders of the Spike mask. A minimum of 20 spike-positive cells were analysed per condition.

Flow cytometry

Cells were harvested and detached with Trypsin-EDTA (Thermo Fisher Scientific), stained with Zombie NIR live-dead dye (Biolegend) in PBS (1:500, 5min), washed and fixed with 4% formaldehyde (Sigma) for 20-30 min at RT. Cells infected with live SARS-CoV-2 virus were fixed with 4% formaldehyde for 1h at RT. Cells were then washed and permeabilised with Perm buffer (Biolegend) for 5 min at RT and stained with primary antibodies (20 min at RT): anti-HA-tag-APC (Biolegend, 16B12, 1:150), anti-Gag-PE

(Beckam Coulter, KC57, 1:250). SARS-CoV-2 infection was detected by intracellular staining for nucleocapsid (N) protein using anti-SARS-CoV N monoclonal antibody (human, clone CR3009, gift from Laura McCoy, UCL, 1 μg/ml) and secondary anti-Human IgG-AF488 (Jackson labs, 1:400). For analysis of cell surface spike infection, 293T cells were harvested with 5 mM EDTA (Sigma), washed, stained with Zombie NIR and anti-SARS-CoV-2 Spike antibody from human convalescent sera (NIBSC, 20/130 1:200) on ice for 30 min. Primary antibodies were detected with the secondary anti-Human IgG-AF488 before fixation and intracellular stain as described above.

SI references

1. L. G. Thorne *et al.*, Evolution of enhanced innate immune evasion by SARS-CoV-2. *Nature* **602**, 487-495 (2022).
2. J. Schindelin *et al.*, Fiji: an open-source platform for biological-image analysis. *Nat Methods* **9**, 676-682 (2012).

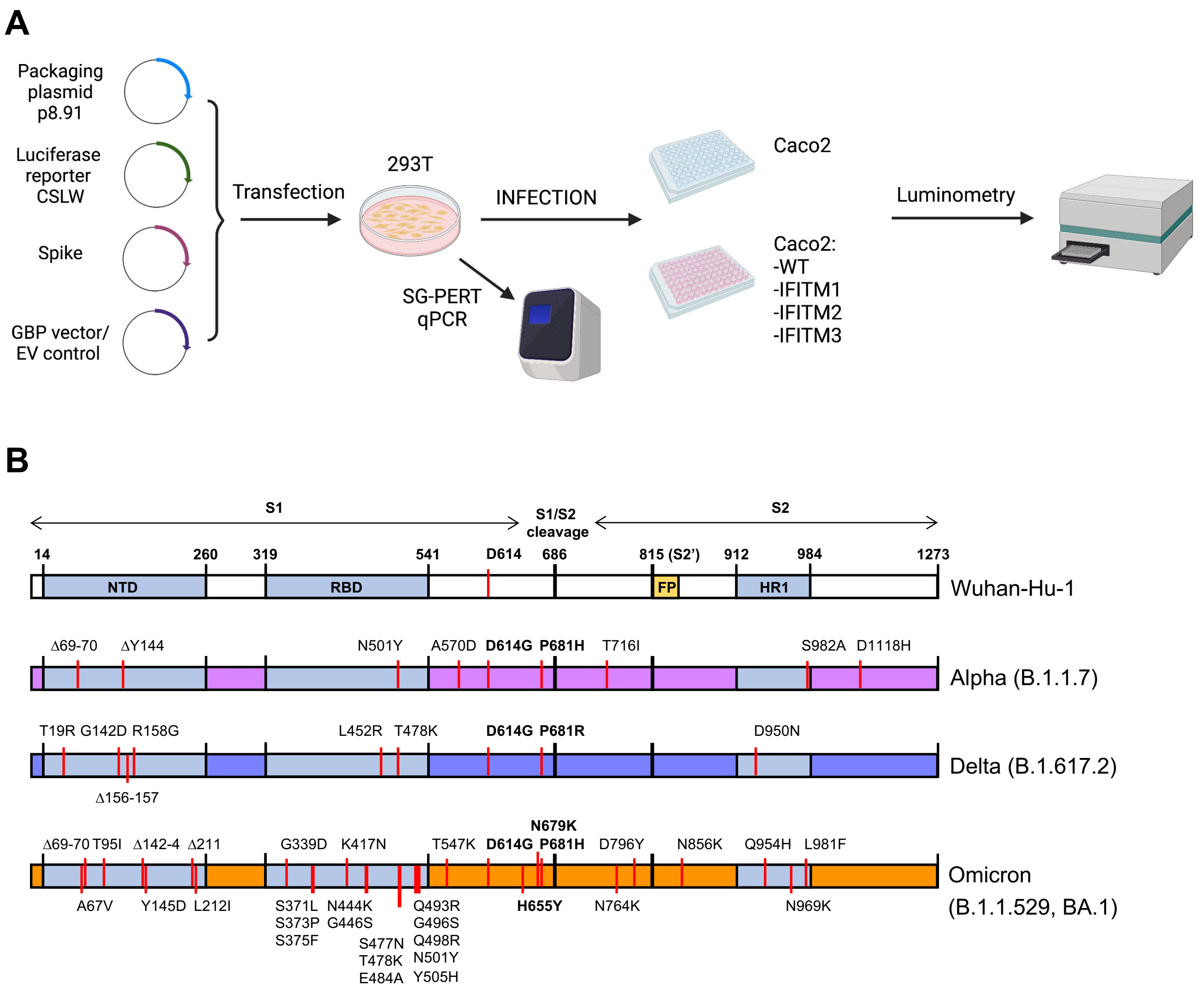

Supplementary Figure 1: Schematic of the pseudovirus infection assay and SARS-CoV-2 spike subunit structure with VOC lineage defining mutations

A) Schematic of the pseudovirus (PV) infection assay. 293T cells were co-transfected with lentiviral packaging, luciferase reporter and spike plasmids (to make PV) and GBP vector or empty vector (EV) control. PV-containing supernatant was used to infect indicated cells lines and infection was quantified by measuring luciferase expression by luminometry. PV content in the supernatants was measured by SG-PERT qPCR assay. Created with BioRender.com **B)** The diagram shows spike subunits and main domain boundaries, with positions of S1/S2 and S2' cleavage sites. Linage defining mutations for Alpha, Delta and Omicron BA.1 VOCs are indicated. NTD, N-terminal domain; RBD, receptor-binding domain; FP, fusion peptide; HR1, heptad repeat 1.

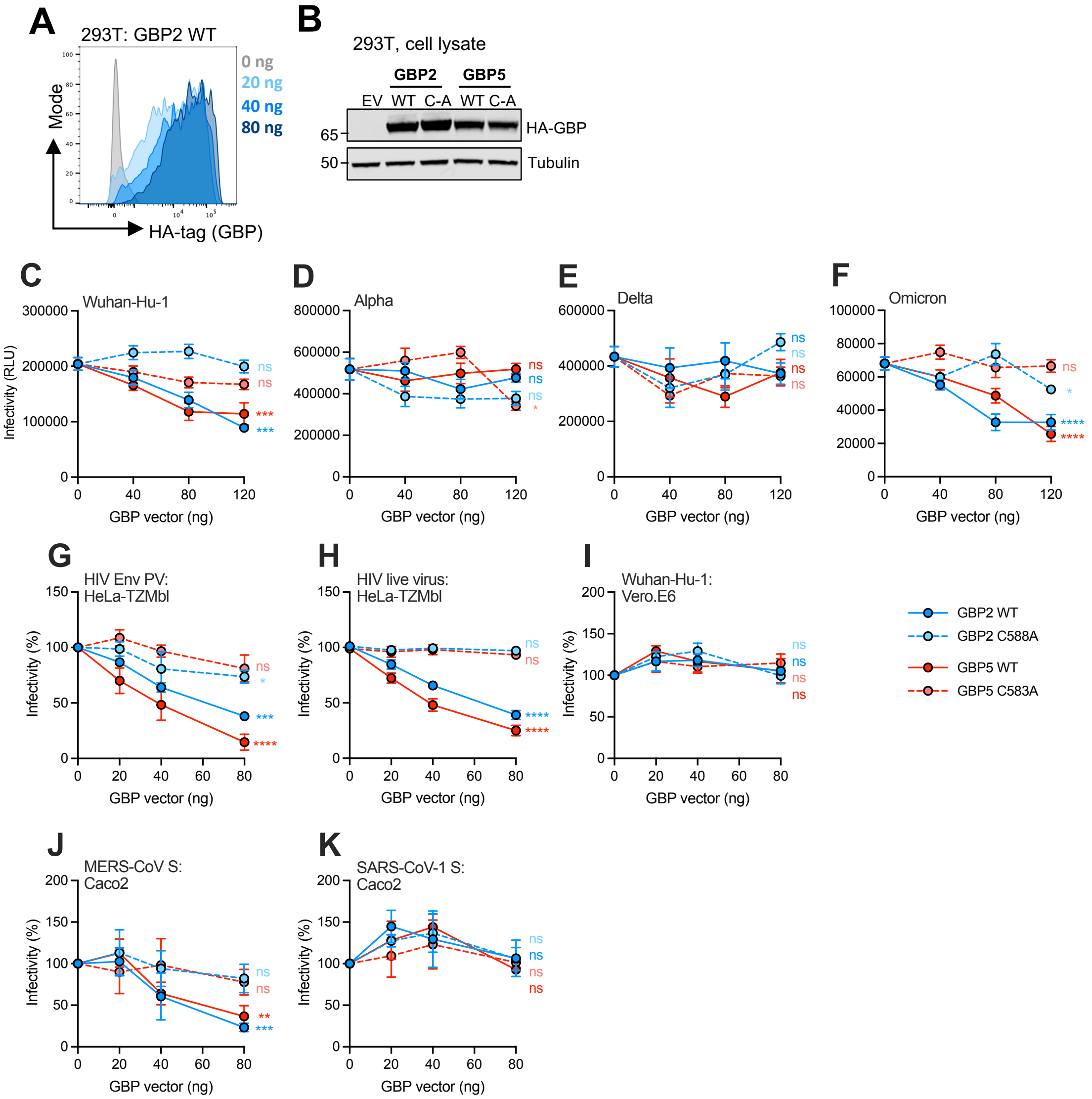

Supplementary Figure 2: GBP restriction of HIV-1 Env, SARS-CoV-2, SARS-CoV-1 and MERS-CoV spike-mediated infection A) Expression of HA-tagged GBP2 in PV-producing 293T cells measured by flow cytometry. Shown is a representative histogram, related to Fig. 1A. **B)** PV-producing 293T cell lysates were immunoblotted for expression of indicated HA-tagged GBPs: GBP2 (or C588A mutant, C-A) and GBP5 (or C583A mutant, C-A) or EV control. **C-F)** Shown are raw RLU values (not normalised to input RT units) from a representative experiment shown in Fig. 1B-E. PV were produced by 293T cells in the presence of increasing amounts of indicated GBPs or EV control. Shown is infectivity (RLU) on Caco2 cells of **C)** Wuhan-Hu-1, **D)** Alpha, **E)** Delta, and **F)** Omicron PVs. Shown are the mean ±SD of three technical replicated from one experiment. *(Continued on the next page)*

(Supp. Fig. 2 continued)

G) HIV Env (JRFL) PV or **H)** HIV-1 live virus (NL4.3) were produced in 293T cells in presence of increasing amounts of plasmid encoding indicated GBPs or EV control. Infection was measured by luciferase assay (RLU) on HeLa-TZMbl reporter cells. Percentage infectivity of PV made in the presence of GBPs normalised to EV control (no GBP, set to 100%) are shown. **I-K)** Spike PV were produced in presence of increasing doses of GBP2/5, GBP mutants or EV control and infection was measured on indicated cell lines. Percent infectivity was normalised to EV control for **I)** SARS-CoV-2 Wuhan-Hu-1 spike PV titrated on Vero.E6 cells, **J)** MERS-CoV spike PV titrated on Caco2 cells, and **K)** SARS-CoV-1 spike PV titrated on Caco2. Shown is mean ±SEM from three independent experiments**.** Two-way ANOVA with Dunnett's post-test was used. Asterisks denote statistical significance for GBPs (80 or 120 ng) compared EV control. ns, not significant; *$p < 0.05$; **$p < 0.01$; ***$p < 0.001$; ****$p < 0.0001$.

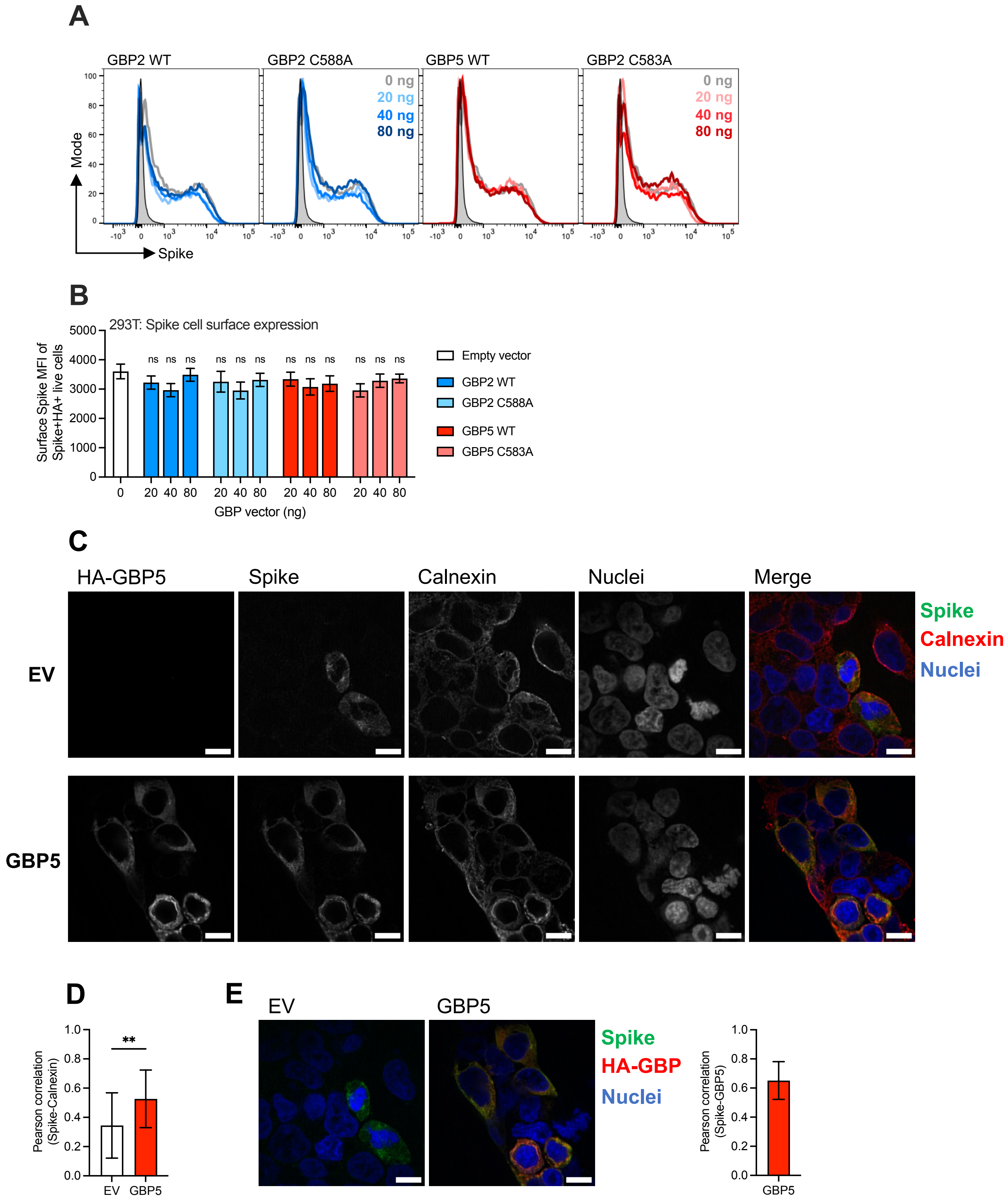

Supplementary Figure 3: The effect of GBPs on the SARS-CoV-2 spike cell surface expression and intracellular localisation *(Continued on the next page)*

(Supp. Fig. 3 continued)

A-B) 293T cells were transfected with Wuhan-Hu-1 spike, lentiviral packaging and reporter plasmids and increasing doses of GBP2/5 or GBP mutants. Cells were stained for cell surface expression of spike and intracellular expression of HA-tagged GBPs. **A)** Shown are representative histograms of spike expression in HA+ live cell population. **B)** Cell surface expression of spike (MFI) in the Spike+HA+ live cell population. Shown is mean ±SEM from three independent experiments. Different GBP treatments were compared to EV control using a RM one-way ANOVA with Dunnett's post-test. **C-E)** 293T cells were transfected with Wuhan-Hu-1 spike, lentiviral packaging and reporter plasmids and 80 ng GBP5 or empty vector. Cells were stained for intracellular expression of spike, HA-tagged GBP5 and calnexin (ER marker), and imaged by confocal microscopy. **C)** Colocalization of spike (green), calnexin (red) and nuclei (blue). The scale bar is 10 µm. **D)** Pearson correlation coefficient of spike vs. calnexin colocalisation, n = 20 cells. Two-tailed t test was used. **E)** Colocalisation of spike (green) and HA-GBP5 (red), (nuclei blue) with Pearson correlation coefficient of spike vs. GBP5 colocalisation (right-hand panel). ns, not significant; *p < 0.05; **p < 0.01.

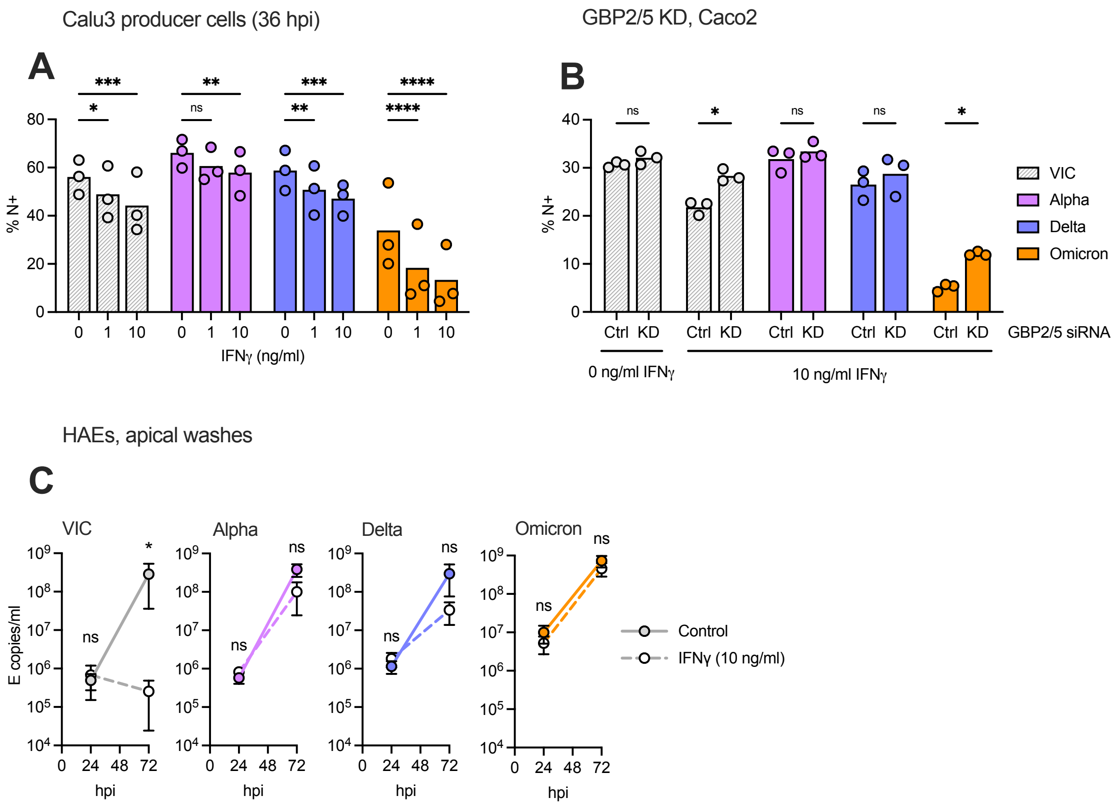

Supplementary Figure 4: IFNγ treatment of Calu-3 and primary human airway epithelial cells (HAEs) inhibits infection and virus infectivity

A) Calu-3 cells were pre-treated with indicated doses of IFNγ for 8 h and infected with indicated SARS-CoV-2 virus isolates for 36 h as described in Fig. 1. Infection was measured by flow cytometry analysis to quantify N expression shown as % N+ cells. Mean and individual values from three independent experiments are shown. **B)** Replicate experiment as shown in Fig. 1J. Calu-3 cells were pre-treated with combined *GBP2* and *5* siRNA (KD) or non-targeting control (Ctrl, 120 and 32 h pre-infection) and indicated doses of IFNγ (8 h pre-infection) before infection with indicated SARS-CoV-2 variants for 36 h. Equal doses (E copies/cells) of virus produced from IFNγ-treated Calu-3 cells were used to infect Caco2 cells or 24 h and infection was quantified by N protein staining. Bars show the mean and replicate values from one experiment.

C) HAEs were pre-treated with indicated doses of IFNγ for 12 h and infected with equal doses of indicated SARS-CoV-2 virus isolates for 72 h as described in Fig. 1. Virus supernatant was collected from the apical washes at 24 and 72 hpi to measure viral release (E copies/ml). Shown is mean ±SEM from 6 measurements from two independent experiments. Two-way ANOVA with Dunnett's post-test (A-B) or Kruskal-Wallis test with Dunn's post-test (C) was used. ns, not significant; *$p < 0.05$; **$p < 0.01$; ***$p < 0.001$; ****$p < 0.0001$.

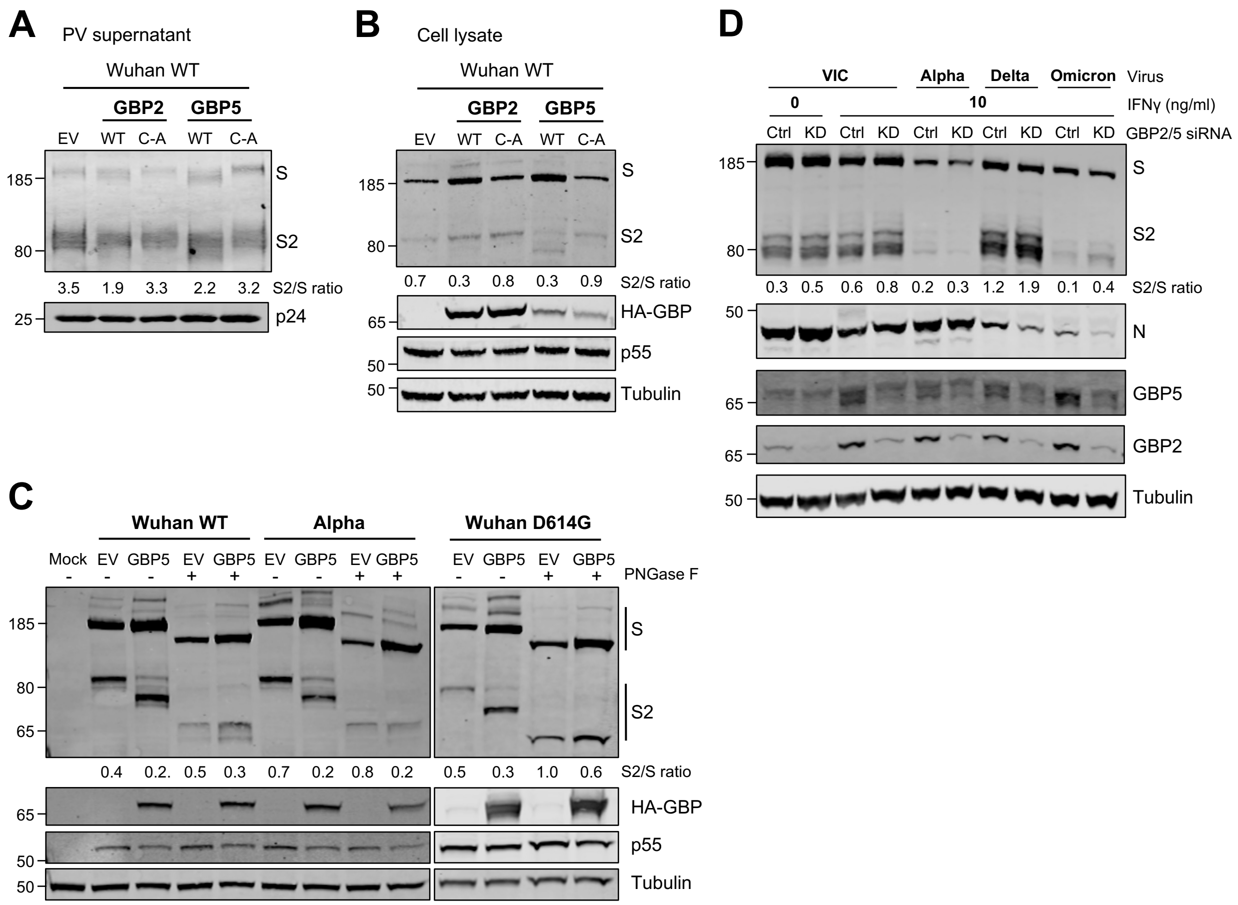

Supplementary Figure 5: GBP2/5 and SARS-CoV-2 spike cleavage and glycosylation

A-B) Immunoblot of Wuhan-Hu-1 spike from PV produced by 293T cells made in presence of 80 ng GBP2 (or C588A mutant, C-A) and GBP5 (or C583A mutant, C-A) or EV control. **B)** Producer 293T cell lysate from (A). PVs and producer cell lysates were immunoblotted for spike (S), lentiviral Gag (p24 and p55), GBP (HA-tag) and tubulin. Quantification shows spike cleavage (S2/S ratio). **B)** Producer 293T cell lysate from (A). **C)** Indicated spike PV were produced in presence of 80 ng GBP5 or EV control by 293T cells. Producer cell lysates were treated with PNGase F (removing N-linked glycans) or left untreated and immunoblotted for spike (S), GBP5 (HA-tag), lentiviral Gag (p55), and tubulin. Quantification shows spike cleavage (S2/S ratio). **D)** Calu-3 cells were pretreated with combined *GBP2* and *5* siRNA (KD) or non-targeting control (Ctrl) and indicated doses of IFNγ before infection with indicated SARS-CoV-2 variants as described in Fig. 1I-C. Cell lysates were immunoblotted for expression of spike (S), nucleocapsid (N), GBP2/5 and tubulin. Same immunoblots of GBP2/5 and tubulin are shown Fig. 1I. Quantification shoes proportion of cleaved spike (S2/S ratio). Corresponding virus supernatant blot is shown in Fig. 2C.

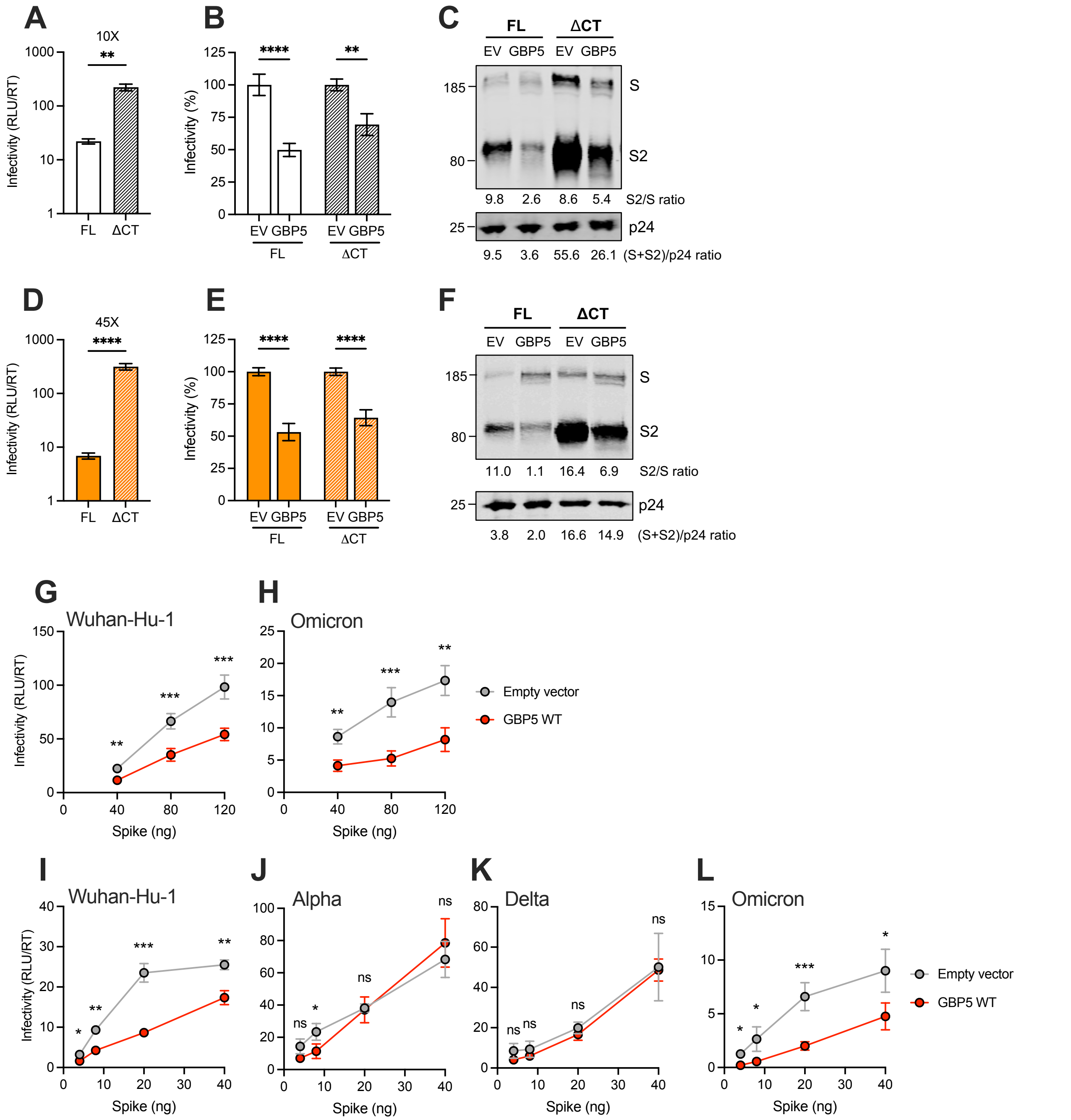

Supplementary Figure 6: C-terminal truncation or increasing expression of Wuhan-Hu-1 and Omicron spike does not rescue from GBP restriction

A-C) Wuhan WT spike PV were produced in presence of GBP5 or EV control using plasmids encoding either full-length (FL) or a 19 residue C-terminal truncation (ΔCT) Wuhan spike sequence. Spike PV were titrated on Caco2 cells, showing **A)** raw infectivity (RLU/RT) values in absence of GBP5 expression (EV control) and **B)** percent infectivity normalised to EV control for each Wuhan spike (FL and ΔCT). *(Continued on the next page)*

(Supp. Fig. 6 continued)

C) Wuhan FL and ΔCT spike PVs produced in the presence of GBP5 or EV control were purified from culture supernatants and immunoblotted for spike and lentiviral Gag (p24). Band quantification shows relative proportion of cleaved spike in PV (S2/S) and cleaved spike incorporation into PV (S2/p24). **D-F)** Omicron spike PV were produced in presence of GBP5 or EV control using plasmids encoding either FL or ΔCT Omicron spike. Spike PV were titrated on Caco2 cells, showing **D)** raw infectivity (RLU/RT) values in absence of GBP5 expression (EV control) and **E)** percent infectivity normalised to EV control for each Omicron spike (FL and ΔCT). **F)** Indicated Omicron spike PVs were immunoblotted for spike and lentiviral Gag (p24). Quantification shows relative proportion of cleaved spike in PV (S2/S) and total spike incorporation into PV (S+S2/p24). **G-L)** Spike PVs were produced with fixed amount of GBP5 vector or EV control (80 ng) and varying doses of spike plasmid and titrated on Caco-2 cells. **G-L)** PV infectivity (RLU/RT) at increasing doses of **G)** Wuhan-Hu-1 and **L)** Omicron spike vector. **I-L)** PV infectivity (RLU/RT) at decreasing doses of **I)** Wuhan-Hu-1, **J)** Alpha, **K)** Delta, and **L)** Omicron spike vector. Bars and data points show mean ±SEM from three independent experiments. Two-tailed t test (A,D), or two-way ANOVA with Dunnett's post-test (B,E, G-L) were used. ns, not significant; *p < 0.05; **p < 0.01; ***p < 0.001; ****p< 0.0001.

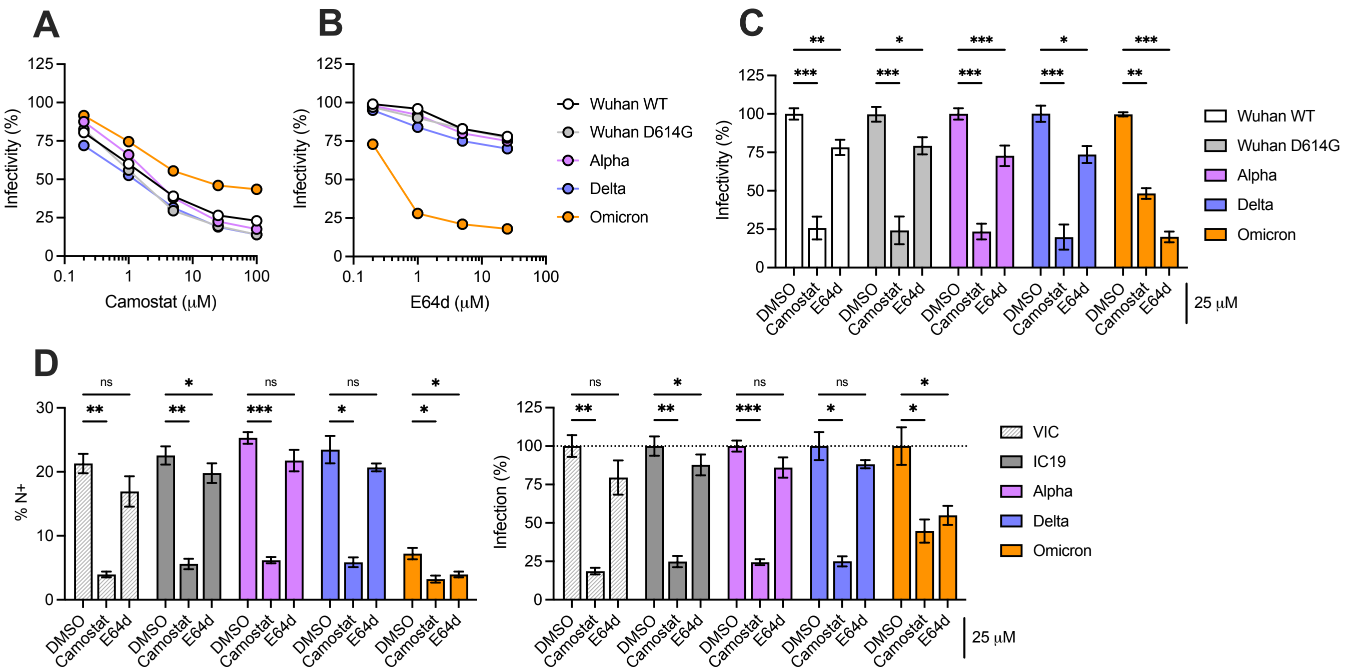

Supplementary Figure 7: Spike-mediated entry pathways in Caco2 cells

Inhibition of spike-mediated PV infection of Caco2 cells by **A)** Camostat and **B)** E64d inhibitors. Shown is a representative titration. **C)** Inhibition of PV infection of Caco2 cells in the presence of 25 µM Camostat or E64d normalised to DMSO control. Infection was measured by luciferase assay. **D)** Inhibition of SARS-CoV-2 live virus infection of Caco2 cells by Camostat or E64d. Infection was measured by at 24 h by intracellular flow cytometry staining for nucleocapsid (N) protein. Percentage N+ cells (% N+) (left) and percentage infection for each virus normalised to the corresponding DMSO control (right) are shown. Mean ±SEM from three independent experiments are shown. Two-way ANOVA with Dunnett's post-test was used. ns, not significant; *p < 0.05; **p < 0.01; ***p < 0.001; ****p < 0.0001.

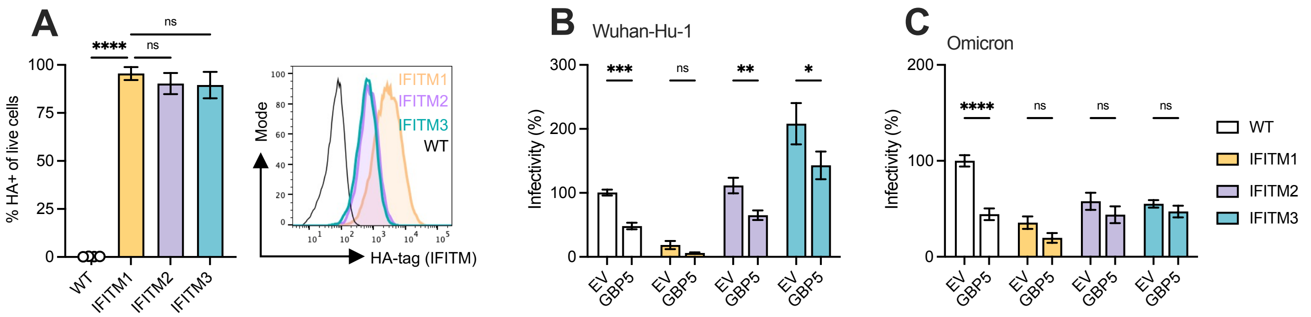

Supplementary Figure 8: GBP5 restriction does not influence IFITM sensitivity

A) Stable expression of HA-tagged IFITMs 1-3 in Caco2 cells measured by flow cytometry. Representative histogram is shown (right). **B-C)** IFITM-expressing Caco2 cells were infected with spike PVs made in presence of GBP5 or EV control. Infectivity normalised to EV control on WT cells for PV made using **B)** Wuhan-Hu-1 and **C)** Omicron spikes is shown.

Bars show mean ±SEM from three independent experiments. One-way ANOVA (A) or two-way ANOVA (B-C) with Dunnett's post- test was used. ns, not significant; *$p < 0.05$; **$p < 0.01$; ***$p < 0.001$; ****$p < 0.0001$.

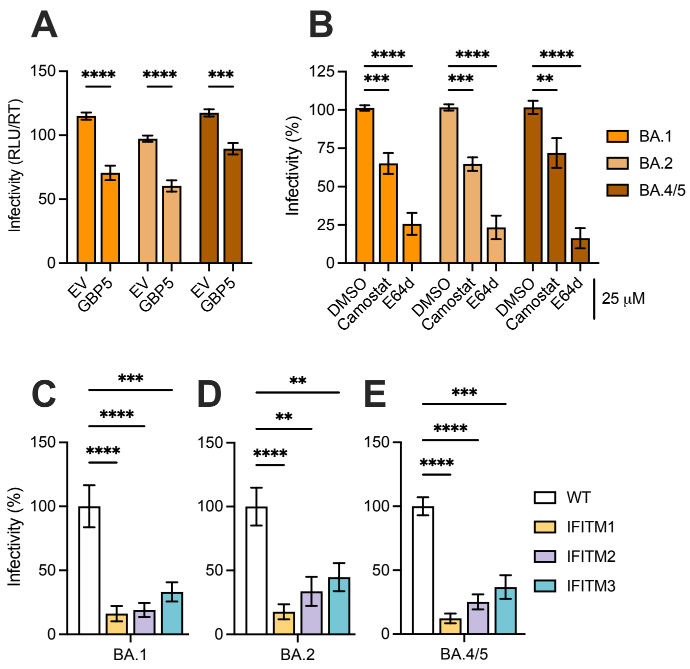

Supplementary Figure 9: Omicron subvariants BA.1, BA.2 and BA.4/5 spike PV are equally sensitive to GBP5, entry inhibitors and IFITMs

A) Omicron BA.1, BA.2 and BA.4/5 ΔCT spike PV were produced in presence of GBP5 or EV control and titrated on Caco2 cells. Shown are raw infectivity (RLU/RT) values. **B)** Inhibition of spike PV infection of Caco2 cells in the presence of 25 µM Camostat or E64d normalised to DMSO control. **C-E)** PV infection of IFITM transduced Caco2 cells with **C)** BA.1, **D)** BA.2, and **E)** BA.4/5 spike PV. Data are shown as percent infectivity normalised to WT Caco2 cells (no IFITM over-expression). Bars show mean ±SEM from three independent experiments. Two-way ANOVA (A, B) or one-way ANOVA (C-E) with Dunnett's post-test were used. ns, not significant; *$p < 0.05$; **$p < 0.01$; ***$p < 0.001$; ****$p < 0.0001$.
